# Poly-glycine–alanine exacerbates *C9orf72* repeat expansion-mediated DNA damage via sequestration of phosphorylated ATM and loss of nuclear hnRNPA3

**DOI:** 10.1007/s00401-019-02082-0

**Published:** 2019-10-23

**Authors:** Yoshihiro Nihei, Kohji Mori, Georg Werner, Thomas Arzberger, Qihui Zhou, Barham Khosravi, Julia Japtok, Andreas Hermann, Andreas Sommacal, Markus Weber, German Consortium for Frontotemporal Lobar Degeneration, Bavarian Brain Banking Alliance, Frits Kamp, Brigitte Nuscher, Dieter Edbauer, Christian Haass

**Affiliations:** 1 German Center for Neurodegenerative Diseases (DZNE) Munich, 81377 Munich, Germany; 2 Department of Psychiatry, Osaka University Graduate School of Medicine, Yamadaoka 2-2 D3, Suita, 565-0871 Osaka Japan; 3 Metabolic Biochemistry, Biomedical Center (BMC), Faculty of Medicine, Ludwig-Maximilians-Universität München, 81377 Munich, Germany; 4 Center for Neuropathology and Prion Research, Ludwig-Maximilians-Universität München, 81377 Munich, Germany; 5 Department of Psychiatry and Psychotherapy, Ludwig-Maximilians Universität München, 80336 Munich, Germany; 6 Department of Neurology, Technische Universität Dresden, 01307 Dresden, Germany; 7 Translational Neurodegeneration Section “Albrecht-Kossel”, Department of Neurology and Center for Transdisciplinary Neurosciences Rostock (CTNR), University Medical Center Rostock, University of Rostock, 18147 Rostock, Germany; 8 German Center for Neurodegenerative Diseases (DZNE) Rostock/Greifswald, 18147 Rostock, Germany; 9 Institute for Pathology, Kanstonsspital St. Gallen, Rorschacher Strasse 95, 9007 St. Gallen, Switzerland; 10 Muskelzentrum/ALS Clinic, Kantonsspital St. Gallen, Rorschacher Strasse 95, 9007 St. Gallen, Switzerland; 11 Munich Cluster for System Neurology (SyNergy), 81377 Munich, Germany

**Keywords:** Amyotrophic lateral sclerosis, *C9orf72*, DNA damage, Frontotemporal lobar degeneration, Heterogeneous ribonucleoprotein A3, Neurodegeneration

## Abstract

Repeat expansion in *C9orf72* causes amyotrophic lateral sclerosis and frontotemporal lobar degeneration. Expanded sense and antisense repeat RNA transcripts in *C9orf72* are translated into five dipeptide-repeat proteins (DPRs) in an AUG-independent manner. We previously identified the heterogeneous ribonucleoprotein (hnRNP) A3 as an interactor of the sense repeat RNA that reduces its translation into DPRs. Furthermore, we found that hnRNPA3 is depleted from the nucleus and partially mislocalized to cytoplasmic poly-GA inclusions in *C9orf72* patients, suggesting that poly-GA sequesters hnRNPA3 within the cytoplasm. We now demonstrate that hnRNPA3 also binds to the antisense repeat RNA. Both DPR production and deposition from sense and antisense RNA repeats are increased upon hnRNPA3 reduction. All DPRs induced DNA double strand breaks (DSB), which was further enhanced upon reduction of hnRNPA3. Poly-glycine–arginine and poly-proline-arginine increased foci formed by phosphorylated Ataxia Telangiectasia Mutated (pATM), a major sensor of DSBs, whereas poly-glycine–alanine (poly-GA) evoked a reduction of pATM foci. In dentate gyri of *C9orf72* patients, lower nuclear hnRNPA3 levels were associated with increased DNA damage. Moreover, enhanced poly-GA deposition correlated with reduced pATM foci. Since cytoplasmic pATM deposits partially colocalized with poly-GA deposits, these results suggest that poly-GA, the most frequent DPR observed in *C9orf72* patients, differentially causes DNA damage and that poly-GA selectively sequesters pATM in the cytoplasm inhibiting its recruitment to sites of DNA damage. Thus, mislocalization of nuclear hnRNPA3 caused by poly-GA leads to increased poly-GA production, which partially depletes pATM, and consequently enhances DSB.

**Electronic supplementary material:**

The online version of this article (10.1007/s00401-019-02082-0) contains supplementary material, which is available to authorized users.

## Introduction

*C9orf72* repeat expansion is the most common cause of autosomal dominant FTLD, FTLD/ALS, and ALS [14, 20, 50]. While unaffected people generally have less than 30 (G_4_C_2_)*n* repeats, mutation carriers have a few hundred or even thousands of repeats [50]. Sense and antisense repeat RNAs accumulate within intranuclear RNA foci [14]. Furthermore, sense and antisense transcripts are translated in all reading frames into dipeptide-repeat proteins (DPRs) in an AUG-independent manner [2, 43]. Accumulating evidence suggests that neurotoxicity occurs via various cellular pathways, such as RNA mis-splicing and reduced transcription of the *C9orf72* gene [27, 29], nucleocytoplasmic transport dysfunction [19, 28, 65, 66], nucleolar stress [23, 38, 60], and DNA damage [15, 32, 58].

We previously identified the heterogeneous ribonucleoprotein (hnRNP) A3 as an interactor of the sense repeat RNA. We and others also found that hnRNPA3 is mislocalized from the nucleus to the cytoplasm specifically in hippocampal, cerebellar, and spinal motor neurons of *C9orf72* patients [17, 41]. Moreover, mislocalized hnRNPA3 colocalizes with poly-glycine-alanine (poly-GA) deposits [42]. Reduction of nuclear hnRNPA3 increases (G_4_C_2_) repeat RNA foci. Furthermore, repeat RNA foci and DPRs may enhance nucleocytoplasmic transport dysfunction, reducing nuclear hnRNPA3 and thus initiating a vicious cycle [42]. Thus, reduction of nuclear hnRNPA3 may be specifically associated with *C9orf72*-dependent neurotoxicity, though the cellular mechanisms remain unclear. There is evidence that *C9orf72*-linked neurotoxicity is driven by genomic instability [58]. Moreover, impairment of DNA damage repair is implicated in several other neurodegenerative diseases, such as Alzheimer disease, Parkinson’s disease, and Huntington disease [6, 8, 9, 11, 21, 25, 33, 34, 36, 64]. DNA double strand breaks (DSBs) are the most severe type of DNA damage and frequently lead to cell death if not correctly repaired. Homologous recombination (HR) and non-homologous end joining (NHEJ) are the two major cellular mechanisms that repair DSBs in mammalian cells. HR fully restores the original sequence using the sister chromatid as a template, but is, therefore, limited to the late S to G2/M phase of the cell cycle [37, 61]. NHEJ is a rather error-prone repair mechanism that rejoins broken DNA ends without a template DNA and can, therefore, occur throughout the cell cycle [47]. Since neurons are arrested in the G0 phase and are non-proliferative, their main repair mechanism is limited to NHEJ. Due to their error-prone repair pathway, neurons are quite vulnerable to DNA damage. This may be particularly important for expanded *C9orf72* repeats, as they form G-quadruplex structures and promote the formation of RNA:DNA hybrids (R-loops) [18, 23, 63], which are prone to DSBs. Walker et al. reported that expanded hexanucleotide repeats and poly-GA impair Ataxia Telangiectasia Mutated (ATM)-mediated DNA repair [58]. Moreover, reduced expression of hnRNPA3 itself may enhance DSBs [13]. Furthermore, many hnRNPs, which are genetically associated with FTD/ALS, such as hnRNPA1, A2B1, and FUS (hnRNPP2) are reported to be involved in DNA damage and repair [4, 12, 24, 44, 48, 55], and hnRNPA3 is a homolog of hnRNPA1 and A2B1 [9, 59]. We speculated that cytoplasmic mislocalization of hnRNPA3 may affect ATM-mediated DNA damage directly [13] via increased repeat RNA foci and DPR production. We now investigated the association of hnRNPA3 expression, RNA foci formation, DPR production, and DNA damage in cultured cells, including patient-derived human neurons and brains of *C9orf72* carriers. Our findings suggest that the most frequent DPRs (poly-GA) observed in *C9orf72* patients differentially cause DNA damage by selectively sequestering phosphorylated ATM (pATM) in the cytoplasm and inhibiting its recruitment to sites of DNA damage.

## Materials and methods

### DNA synthesis and plasmid construction for in vitro transcription

We synthesized the plasmid containing hexanucleotide repeats for in vitro transcription by using a previously reported protocol [41]. In brief, 124 base single-stranded DNA containing G_4_C_2_, C_4_G_2_, or A_4_C_2_ hexanucleotide repeats with restriction enzyme sites (NheI or HindIII) were synthesized (Suppl. Fig. 1a). 100 μM of complementary DNA strands were annealed in the presence of 10% GC-RICH solution (Roche) and GC-RICH PCR Reaction buffer (Roche) and cloned into pcDNA3.1(+) vector (Invitrogen). Plasmids containing 17 repeats of G_4_C_2_, C_4_G_2_, and A_4_C_2_ were obtained. The DNA sequence of all constructs was verified.

### In vitro transcription of RNA probes

pcDNA3.1-(G_4_C_2_)17, pcDNA3.1-(C_4_G_2_)17, and pcDNA3.1-(A_4_C_2_)17 constructs were linearized with HindIII and used as templates for RNA synthesis (Suppl. Fig. 1a). In vitro RNA transcription was performed with T7 Ribomax Express Large Scale RNA Production System (Promega) supplemented with 40 U of RNase inhibitor (RiboLock, Thermo Scientific) as described by the manufacturer. To achieve equal levels of biotinylation between these probes, different concentrations of biotin-14-CTP (1 mM for G_4_C_2_ probe, 0.08 mM for C_4_G_2_ probe, and 1.2 mM for A_4_C_2_ probe) were added in each reaction. Following DNase treatment, biotinylated RNA products were purified with phenol/chloroform. For competition experiments non-biotinylated C_4_G_2_ repeat RNA was in vitro RNA transcribed using the MEGA script kit (Ambion) as described by the manufacturer. The expected lengths of repeat RNA probes and competitor were confirmed by formaldehyde gel electrophoresis (Suppl. Fig. 1b). Biotinylation efficacy of RNA probes was evaluated using the BrightStar BioDetect kit (Ambion) (Suppl. Fig. 1c).

### Purification of hexanucleotide repeat-binding proteins

Purification of hexanucleotide repeat-binding proteins was performed following our previously described protocol [41]. In brief, a total of 0.6 mg of HeLa cell nuclear extract was diluted in 4 ml of protein-binding buffer (10% glycerol, 10 mM HEPES, 50 mM KCl, 1 U/ml RNase inhibitor, 0.15 lg/ml yeast tRNA (10109495001, Roche), 1 mM EDTA, 1 mM DTT and 0.5% Triton X100 in DEPC water). The diluted extract was then precleared with heparin-agarose (H6508, Sigma-Aldrich) and streptavidin-agarose (15942-050, Invitrogen). The precleared nuclear extracts were incubated with streptavidin μMACS-microbeads (MACS molecular) and 150 pmol of biotinylated repeat RNA in the presence of 50 mM KCl for 1 h. For competition experiments, nuclear extracts were incubated for 1 h with 7.5 nmol (50-fold excess) of non-biotinylated (C_4_G_2_)17 competitor RNA before addition of the biotinylated probe. The reaction mixture was loaded on a μMACS column and subsequently washed three times with protein-binding buffer. Hexanucleotide repeat binding proteins were sequentially eluted with increasing concentrations of NaCl. Each eluate was TCA precipitated and subjected to SDS-PAGE.

### Cell culture

HeLa cells were cultured in DMEM containing 10% FCS and Penicillin/Streptomycin at 37 °C with 5%CO_2_.

### Patient-derived fibroblasts

We included cell lines from 3 *C9orf72* ALS patients and 3 control cases from our previous report [42]. All procedures were in accordance with the Helsinki convention and approved by the Ethical Committee of the University of Dresden (EK45022009; EK393122012). Patients were genotyped using EDTA blood in the clinical setting after given written consent according to German legislation independent of any scientific study by a diagnostic human genetic laboratory (CEGAT, Tübingen, Germany or Department of Human Genetics, University of Ulm, Germany) using diagnostic standards.

For further details of patients’ background, see Suppl. Table 1.

### siRNA-mediated knockdown in fibroblasts and iPSC-derived neurons

The following siRNAs were obtained from Dharmacon: ON-TARGETplus human non-targeting siRNA D-001810-01, hnRNPA3 J-019347-08 ACAAUGAAGGAGGAAAUUU, hnRNPA3 J-019347-06 GGAGGGAACUUUGGAGGUG. 10nMol of each siRNA was reverse transfected using RNAiMax (Thermo Fisher Scientific) and OPTI-MEM. Media were exchanged after overnight incubation. Cells on coverslips were fixed on the next day (48 h after a transfection of siRNA containing solution).

### Plasmid transfection

For plasmid transfection in HeLa cells, 0.5 μg/well (in the case of 24-well plate) of DNA was transfected with lipofectamine LTX with plus reagent (Thermo Fisher Scientific) in OPTI-MEM. Plasmid containing media were exchanged after 6 h-incubation. Cells were harvested 48 h after plasmid transfection.

### Fluorescence in situ hybridization for antisense RNA foci

Fluorescence in situ hybridization (FISH) was performed as previously described [39, 42, 51] with slight modifications. 2% paraformaldehyde fixed and perforated cells on glass coverslips were rinsed twice with 2 × saline-sodium citrate buffer (SSC) and then incubated in prehybridization solution (40% formamide (Life Technologies, 15515-026)/2 × SSC, 2.5% bovine serum albumin (BSA)) at 57 °C for 30 min. Cells were then incubated with hybridization solution (40% formamide, 2 × SSC, 0.8 mg/ml tRNA (Roche), 0.8 mg/ml single strand salmon sperm DNA (Sigma-Aldrich, D7656), 0.16% BSA, 8% Dextran sulfate (Sigma-Aldrich), 1.6 mM Ribonucleoside vanadyl complex (New England Biolabs, S1402S), 5 mM EDTA, 10 ng/ml 5′ Cy3-labbeled 2′-O-methyl-(CCGGGG) × 4 probe (IDT)) at 57 °C. The following day, cells were sequentially washed in 40% formamide/0.5 × SSC for 3 times 30 min each at 57 °C and then with 0.5 × SSC 3 times 10 min each at room temperature. After a brief rinse with PBS, nuclei were counterstained with 0.5 μg/ml of DAPI for 20 min and then washed with PBS for 3 times 3 min each. Glass coverslips were mounted using Prolong Gold antifade (Life Technologies) and analyzed with LSM710 confocal microscopy with ZEN2011 software (Zeiss). For FISH of fibroblasts, after incubation with hybridization solution and washing, blocking with 5% FCS (30 min) and nucleolin (NCL) staining (4 °C overnight) was performed. Nuclei were counterstained with DAPI and glass coverslips were mounted on the following day.

### Differentiation of human neural precursor cells (NPCs) to spinal motor neurons (MNs)

The generation of all iPSCs was recently published [22, 26, 52]. The generation of human NPCs and MNs was accomplished following the protocol from Naumann et al. [44]. In brief, colonies of iPSCs were collected and stem cell medium, containing 10 µM SB-431542, 1 µM Dorsomorphin, 3 µM CHIR 99021 and 0.5 µM pumorphamine (PMA), was added. After 2 days hESC medium was replaced with basal media (DMEM-F12/Neurobasal 50:50 with 1:200 N2 Supplement, 1:100 B27 lacking Vitamin A and 1% penicillin/streptomycin/glutamine). On day 4 150 µM ascorbic acid was added, while Dorsomorphin and SB-431542 were withdrawn. 2 Days later the EBs were mechanically separated and replated on Matrigel coated dishes. For this purpose, Matrigel was diluted (1:100) in DMEM-F12 and kept on the dishes over night at room temperature. Possessing a ventralized and caudalized character the arising so called small molecule NPCs (smNPC) formed homogenous colonies during the course of further cultivation. It was necessary to split them at a ratio of 1:10–1:20 once a week using Accutase for 10 min at 37 °C.

To identify Hb9-positive neurons, we transduced the Hb9::GFP promoter into the neural progenitor cells using a lentivirus system [30, 45], and the resulting neural progenitors were replated on Poly-L-Ornithine / laminin coated dish and cultured in basal media supplemented with 1 µM PMA. After 2 days 1 µM retinoic acid (RA) was added. On day 9 another split was performed to seed them on a desired cell culture system. Furthermore, the medium was modified to induce neural maturation. For this purpose, the developing neurons were treated with N2B27 containing 10 ng/µl BDNF, 500 µM dbcAMP and 10 ng/µl GDNF. Finally, motor neuron differentiation was confirmed by immunocytochemistry.

### Plasmids

The (G_4_C_2_)_80_ and (C_4_G_2_)_30_ expression vectors are based on a previously published cDNA construct [42]. The modified vector expresses (C_4_G_2_)_30_ under the control of the CMV promoter including 620 bp of the 5′ flanking region of the human *C9orf72* C_4_G_2_ repeat which was PCR subcloned from patient fibroblast derived genomic DNA. This patient has a GGGCCCGCCCCC insertion just before the beginning of the CCCCGG repeat, where frequent sequence variations have been observed [40, 46, 57]. The 5′ flanking region contains multiple stop codons in each reading frame.

Synthetic DPR constructs containing an ATG start codon for transient expressions in HeLa cells were constructed as previously described [7] with slight modifications. Codon optimized DNA sequences encoding 12 repeats of each DPR, were designed. Restriction sites for XhoI-FokI and BbsI-XbaI were placed at the beginning and end of the DNA oligonucleotide. The synthesized DNA oligonucleotide was subcloned into the pEGFP-C1 vector (Clontech) using XhoI and XbaI sites. The codon optimized 11 repeat coding fragments were isolated through double digestion with FokI and BbsI, and then ligated into BbsI site of the parental 12 repeats vector to double the repeat-length. This was repeated until 175–177 repeats were achieved (12 + 11 = 23 repeats, 23 + 22 = 45 repeats, 45 + 44 = 89 repeats, 89 + 86 = 175 repeats (poly-GA), 89 + 88 = 177 repeats (poly-glycine–arginine: poly-GR), 89 + 87 = 176 repeats (poly-proline-arginine: poly-PR). The repeat number differences at the last step are due to clonal instability). The following oligonucleotides were used.

XhoI-FokI-GA12-BbsI-XbaI: 5′-CTCGAGGGATGTTGAATTCTGGTGCTGGCGCGGGAGCAGGCGCTGGTGCTGGTGCAGGAGCGGGTGCGGGAGCTGGTGCCGGCGCAGGTGCTGTCTTCGGATCCTAGTCTAGA-3′.

XhoI-FokI-GR12-BbsI-XbaI: 5′-CTCGAGGGATGTTGAATTCTGGTCGTGGACGTGGACGAGGTCGAGGTCGAGGTCGTGGACGTGGTCGAGGTCGAGGTCGTGGACGTGGTCGTGTCTTCGGATCCTAGTCTAGA-3′.

XhoI-FokI-PR12-BbsI-XbaI: 5′-CTCGAGGGATGTTGAATTCTCCGCGACCTCGACCGCGGCCACGCCCACGCCCTCGGCCCAGACCACGTCCTAGGCCCAGACCCAGACCGCGAGTCTTCGGATCCTAGTCTAGA-3′.

Synthetic DPR constructs containing an ATG start codon for lentiviral expression (synapsin promoter) and transient transfection (EF1 promoter) were described before [9].

### Crispr/Cas9 genome editing

Knockout of hnRNPA3 was performed in HeLa cells using the Crispr/Cas9 [49]. Guide RNA sequences targeting hnRNPA3 genetic loci were designed using the gRNA tool from the Zhang laboratory [https://crispr.mit.edu (Suppl. Fig. 2a)]. Duplexed sgRNA oligos were digested and ligated into pSpCas9(BB)-2A-Puro (PX459) to generate hnRNPA3 Puro plasmid. CRISPR knock-out cells were generated by transfection with hnRNPA3 Puro plasmid followed by selection with 1 µg/ml puromycin. Individual clones were generated by plating cells at low density and isolating individual colonies. hnRNPA3 knockout was confirmed by DNA sequencing (Suppl. Fig. 2b) and western blotting (Fig. 2a). For sequencing of hnRNPA3, genomic loci were amplified by PCR using the following primers:

Forward: 5′-GTATGTCAGCCGCGTTTT-3′, Reverse: 5′- CGGCGGATCAATGTCAAT-3′.

### Western blotting

To detect the protein expression (except for pATM), samples were separated on 12% Tris-glycine gel and transferred on PVDF membranes. After blocking for 1 h with 0.2% I-Block (Applied Biosystems) in TBST (TBS with 0.5% Tween20), membranes were incubated with indicated antibody overnight. The antibody signal was detected with HRP-conjugated secondary antibodies (Promega) using the ECL reagents (GE healthcare) and exposed to X-ray films (SuperRX, Fujifilm).

For detection of pATM, samples were separated on NuPAGE 3–8% Tris-Acetate protein gel (Invitrogen) and transferred on PVDF membrane with 25 V for 15 h. After blocking for 1 h with 5% PhosphoBLOCKER (Cell Biolabs, AKR-104) in TBST, membranes were incubated with indicated antibody for 2 h at room temperature. All subsequent steps were performed as described above.

### Co-immunoprecipitation (co-IP)

HeLa cells were lysed in co-IP buffer (150 mM NaCl, 50 mM Tris–HCl pH 7.4, 0.5% NP-40, 0.5% Na deoxycholate, 5 mM EDTA) supplemented with EDTA-free Protease Inhibitor Cocktail (Roche, 04 693 132 001) and PhosSTOP (Roche, 04 906 845 001), then incubated for 30 min on ice. Lysates were centrifuged for 5 min at 5000 rpm, and the supernatant was collected. For co-IP, the Dynabeads Protein G immunoprecipitation kit (Invitrogen, 10007D) was used. The bead-antibody-antigen complex was isolated using a magnetic tool (Invitrogen, 12321D) and resuspended in elution buffer and NuPAGE LDS sample buffer (Invitrogen, NP0007) supplemented with DTT. The complex was heated for 10 min at 70 °C and captured proteins were separated from bead-antibody complexes using a magnetic tool. Isolated antigens were analyzed by western blotting.

### Cell fractionation

Cell fractionation was performed as previously described [5]. Briefly, cells were washed twice in ice-cold PBS. Cells were harvested in 400 μl buffer 1 (50 mM Tris (pH 7.9), 10 mM KCl, 1 mM EDTA, 0.05% NP-40, 10% glycerol and protease/phosphatase inhibitors) and centrifuged at 6,000 r.p.m. for 3 min at 4 °C. The supernatants were used for the analysis of cytoplasmic proteins. The nuclear pellet was lysed with 150 μl buffer 2 (20 mM HEPES (pH 7.9), 400 mM NaCl, 10 mM KCl, 1% NP-40, 20% glycerol, 1 mM EDTA and protease/phosphatase inhibitors) for 20 min at 4 °C, then centrifuged at 14,000 r.p.m. for 10 min. The supernatants were used for the analysis of nuclear proteins.

### Immunofluorescent staining for HeLa cells and neurons

Cells on glass coverslips were washed with PBS and fixed with 4%PFA for 15 min on ice and permeabilized with 0.2% Triton-X100 in PBS for 5 min at room temperature. After that cover slips were washed with PBS 3 times and blocked with 5%FCS in PBS. Primary antibody incubation was performed overnight at 4 °C, and incubation of secondary antibody conjugated with Alexa Fluor 555 or Alexa Fluor 488 (anti-rabbit, anti-mouse, and anti-rat) was performed for 1 h at room temperature. After a brief rinse with PBS, nuclei were counterstained with 0.5 μg/ml of DAPI for 20 min and then washed with PBS for 3 times 5 min. Finally, coverslips were mounted using Prolong Gold antifade (Life Technologies).

### Quantitative immunofluorescence in HeLa cells and neurons

Three to ten fluorescent images of HeLa cells and cultured neurons were obtained from each glass coverslip using LSM710 (Zeiss) microscope with a 63 × oil immersion objective. Raw data tif files of each channel were exported from czi files using Zen2011 software. Quantification was performed using ImageJ software. The numbers of γH2AX foci positive HeLa cells and iPSC-derived neurons were determined with cell counter plugin of Image J.

### Human brain samples

All cases were provided by the Neurobiobank Munich, Ludwig-Maximilians-University (LMU) Munich, Germany and the ALS Tissue Bank Kantonspital St. Gallen, Switzerland and collected and distributed according to the guidelines of the local ethics committees. Autopsies were performed on basis of informed consent. For further details, see Table 1.

**Table 1:** Demographics of postmortem cases used in the neuropathological study

Case	Case no	Source	Gender	Age at disease onset (years)	Age at death (years)	Clinical diagnosis	Fixation time (days)	Post mortem time (h)
C9	1	Munich	male	45	49	ALS without dementia	98	26
C9	2	Munich	male	45	47	bvFTD	< 59	21
C9	3	Munich	female	68	81	FTD + Depression	287	102
C9	4	St. Gallen	male	71	72	ALS Without dementia	7	8.5
C9	5	Munich	male	67	69	Dementia with Lewy bodies	69	36–60
C9	6	Munich	male	55	61	CBD	210	33
C9	7	Munich	female	50	58	Dementia	n.i	19
C9	8	Munich	male	68	76	Pick's disease (Dementia)	35	130
C9	9	Munich	male	< 64	68	Dementia with Lewy Bodies + Parkinson	42	192
C9	10	Munich	male	60	65	Atypical Parkinsonism + semantic dementia	87	30
C9	11	Munich	female	58	59	Bulbar MND + beginning bvFTD	87	46
C9	12	Munich	male	n.i.	74	FTD	40	37
C9	13	Munich	male	54	57	bvFTD (Pick)	< 135	22
C9	14	Munich	female	60	63	ALS Without dementia	56	37
C9	15	St. Gallen	female	62	66	ALS Without dementia	7	2
C9	16	Munich	female	46	46	ALS Without dementia	19	20
Ct	1	Munich	male	n.a.	61	n.a.	7	10–34
Ct	2	Munich	male	n.a.	46	n.a.	10	10–34
Ct	3	Munich	male	n.a.	62	n.a.	430	46
Ct	4	Munich	male	n.a.	82	n.a.	63	63
Ct	5	Munich	female	n.a.	73	n.a.	111	16
Ct	6	Munich	male	n.a.	74	n.a.	274	33
Ct	7	Munich	male	n.a.	67	n.a.	20	25–31
Ct	8	Munich	male	n.a.	58	n.a.	154	22
Ct	9	Munich	male	n.a.	63	n.a.	4	18

*ALS* amyotrophic lateral sclerosis, *bvFTD* frontotemporal dementia behavioural variant, *CBD* corticobasal degeneration, *FTD* frontotemporal dementia, *MND* motoneuron disease, *n.a.* not applicable, *n.i.* no information

### Immunofluorescent staining for human brain sections

Immunohistochemical staining was performed on 5 μm-thick paraffin sections. Following antigen retrieval by microwaving for 30 min in citrate buffer (10 mM citric acid, 0.05% Tween20, pH 6.2) for co-staining of hnRNPA3 and poly-GA, or citrate-EDTA buffer (10 mM citric acid, 2 mM EDTA, 0.05% Tween20, pH 6.2) for all other stainings and settling the sections at room temperature for 20 min, sections were rinsed with PBS-Tween20 for 2 min, 2 times. After blocking with 2% FCS in PBS for 30 min, sections were incubated in primary antibody overnight at 4 °C. Sections were then rinsed with PBS for 5 min, 3 times, and then incubated in secondary antibody solution for 1 h at room temperature. After rinsing with PBS 2 times, 2.5 min each, nuclei were counterstained with 0.5 μg/ml of DAPI for 20 min and sections were then washed with PBS for 2 times 2.5 min each. To reduce autofluorescence, slides were incubated with 0.3% Sudan Black in 70% ethanol for 1 min. After a rinse with tap water, sections were mounted with using Prolong Gold antifade (Life Technologies).

### Quantitative immunofluorescence in human brain sections

To quantify nuclear hnRNPA3 signal intensity in granular cells of human dentate gyri, we followed the protocol that we previously reported [42]. Fluorescent images of three different areas of hippocampal dentate gyrus granular cell layer at the level of lateral geniculate body were obtained from each case as indicated in Suppl. Fig. 3 using a LSM710 (Zeiss) microscope with a 63 × oil immersion objective. One image was shot for each area. Raw data tif files of each channel were exported from czi files using Zen2011 software. Quantification was performed using ImageJ software. Binary images from DAPI staining were used for the nuclear regions. Vascular structures were manually excluded from the analysis. Granular cell numbers of each image were counted. Nuclear hnRNPA3 was defined as hnRNPA3 signal overlapping with DAPI staining. The hnRNPA3 signal from structures outside of cell bodies was regarded as background. After subtraction of background, nuclear hnRNPA3 intensities of 242–636 granular layer cells/case were quantified in 16 *C9orf72* cases. Median nuclear hnRNPA3 intensities (arbitrary unit) of these cells per micrograph were defined as the nuclear hnRNPA3 level of each image. An average of the nuclear hnRNPA3 levels of 3 micrographs from a *C9orf72* case was regarded as the nuclear hnRNPA3 level of the case. The numbers of γH2AX foci positive cells, poly-GA aggregates positive cells, and pATM foci positive cells were determined by cell counter, the plugin of ImageJ software.

### Filter trap analysis in HeLa cells

Cells cultured in 6-well plates were lysed in 600 μl lysis buffer (25 mM HEPES pH 7.6, 150 mM NaCl, 3% SDS, 0.5% sodium deoxycholate, 1% Triton X-100) supplemented with protease inhibitor cocktail (Sigma-Aldrich) for 10 min and passed through 27G needle for 10 times. The lysates were further diluted to 2 μg or 0.5 μg protein per 200 μl with lysis buffer. 100 μl of each sample were filtered through a nitrocellulose membrane (0.45 μm pore). The membrane was subsequently washed once with TBST, and then blocked in I-Block/TBS/0.5%TritonX-100. Levels of each DPR were analyzed with antibodies against each three different tag (FLAG, myc, HA). Quantified signals from 2 or 3 independent filter trap analyses probed with 3 different tag antibodies (total 6 or 8 membranes) are shown as fold expression.

### Antibodies

The following antibodies were used for Western blotting (WB), immunoprecipitation (IP), and immunofluorescence (IF): anti-hnRNP A1 antibody, clone 4B10 (Sigma-Aldrich, 05-1521) WB 1/8,000, anti-hnRNP A2/B1 antibody, clone DP3B3 (Sigma-Aldrich, R4653) WB 1/10,000, anti-hnRNP A3 antibody, ab1 (Sigma-Aldrich, AV41195, lot No. QC10071) WB 1/2,000 and 1/150 (human brain sections), anti-DYKDDDDK(FLAG) Tag antibody (Cell Signaling #2368S) WB 1/1,000, anti-myc clone 9E10 (Santa Cruz, sc-40) WB 1/1,000, anti-HA Tag antibody, clone 3F10 (Roche, 12158167001) WB 1/1,000, anti-GFP antibody, clone N86/8 (Neuromab) WB 1/3,000, anti-GFP antibody (abcam, ab6556) IF 1/2,000, anti-GFP antibody (Novus Biologicals, NB100-1770) IF 1/200, anti-β-actin antibody (Sigma-Aldrich, A5316) WB 1/2,000, anti-tubulin βIII antibody, clone 2G10 (Millipore, 05-559) WB 1/5,000, IF 1/200, anti-NCL antibody (Abcam, ab136649) IF 1/1,000, anti-phospho-Histone H2A.X (Ser139) antibody, clone JBW301 (Merck Millipore, 05-636) IF 1/250 (for HeLa cells and human brain sections), anti-ATM (phospho S1981) antibody (abcam, ab81292) IP 1/30, WB 1/2,500, anti-ATM (phospho S1981) antibody (abcam, ab36810) IF 1/250, anti-FUS antibody (Bethyl, A300-294A) IF 1/1,500, anti-HuR antibody (SantaCruz, sc-5261) IF 1/100, anti-α-tubulin (Sigma-Aldrich, T5168) WB 1/4,000, anti-Lamin A/C antibody (Cell Signaling, #2032) WB 1/1,000 and anti-GA antibody (Mackenzie et al. [35]) IF 1/500.

### Quantitative reverse transcription PCR (qPCR)

We performed qPCR following the protocol from our previous report [42]. Total RNA was prepared using the RNeasy and Qiashredder kit (Qiagen). RNA preparations were treated with Turbo DNA-free kit (Thermo Fisher Scientific) to minimize residual DNA contamination. Two micrograms of RNA were used for reverse transcription with M-MLV Reverse Transcriptase (Promega) using oligo-(dT) 12–18 primer (Invitrogen). qPCR was performed using the 7500 Fast Real-Time PCR System (Applied Biosystems) with TaqMan technology. Primers and probes were designed (IDT) for the detection of a region flanking the TAG 3′ of the G_4_C_2_ repeat of repeat constructs (repeat TAG primer). Sense repeat, primer 1: TCT CAA ACT GGG ATG CGT AC, primer 2: GTA GTC AAG CGT AGT CTG GG, probe: 5′-/56-FAM/TG CAG ATA T/Zen/C CAG CAC AGT GGC G/3IABkFQ/-3′. Antisense repeat, primer 1: CAA ACC CGG GTA CCC ATA C, primer 2: CGG GCC CTC TAG ACT ACT T, probe: 5′-/56-FAM/ACG TCC CAG/Zen/ACT ACG CTT GAC TAC A/3IABkFQ/-3′. A primer/probe set for Human GAPDH, 4326317E (Applied Biosystems) was used as endogenous control. Each sample was paired with no reverse transcription controls showing < 1/2^10^ (ΔCT > 10) signal when compared to reverse transcribed samples, thus excluding contamination of plasmid DNA-derived signal. Signals from repeat construct derived cDNAs were normalized to GAPDH cDNA according to the ΔΔCT method.

### Statistics

Statistical analysis was performed using IBM SPSS Statistics 23 software. All statistical results are shown in Suppl. Table 2.

## Results

### hnRNPA3 binds to the *C9orf72* antisense repeat RNA

**Fig. 1:**
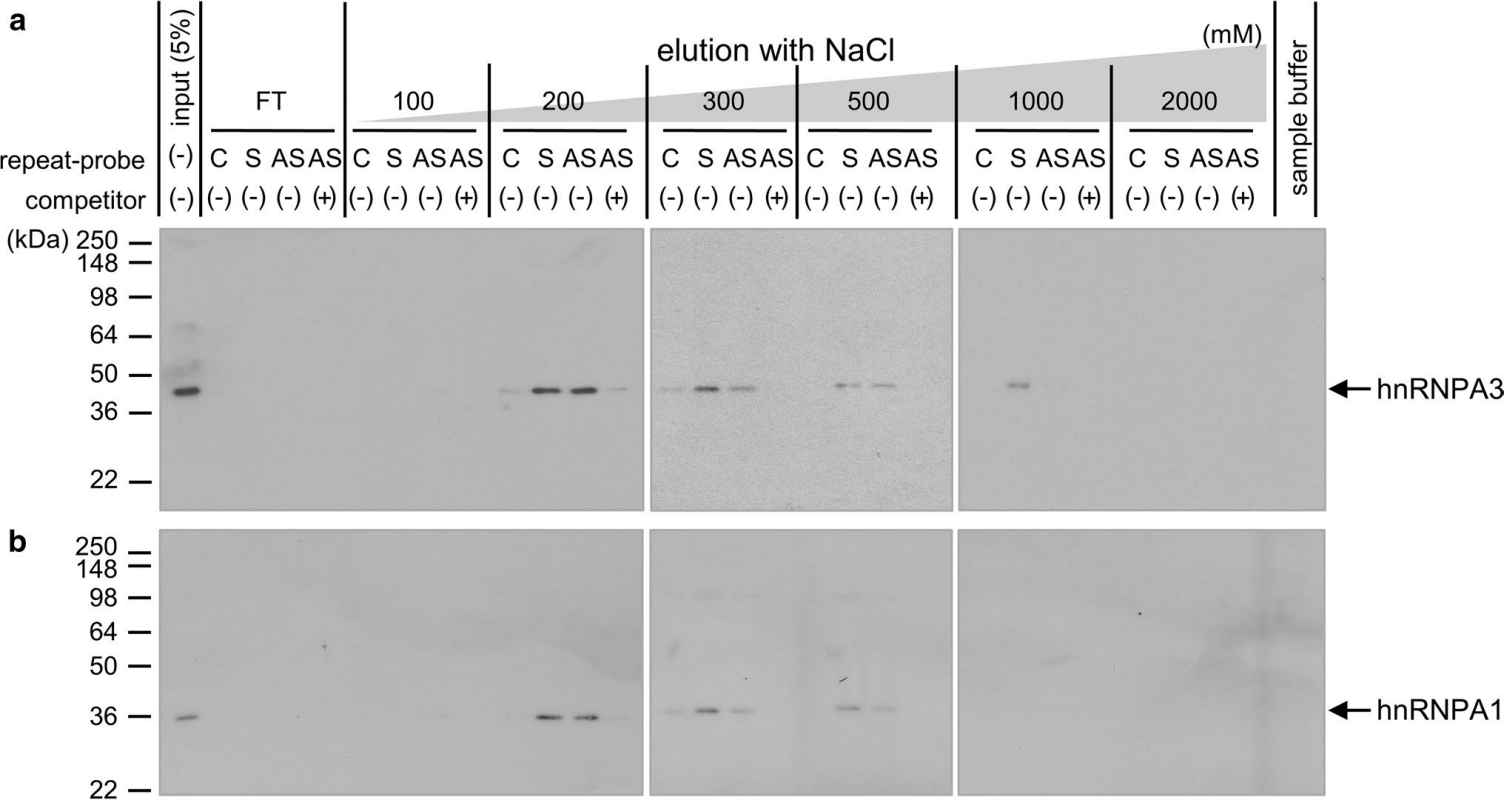
hnRNPA3 and hnRNPA1 bind to sense and antisense repeat RNAs. Nuclear extracts of HeLa cells were incubated with the indicated biotinylated RNA probes with (+) or without (−) 50-fold excess of non-biotinylated RNA competitor and pulled down by magnet beads. **a** Western blotting with an anti-hnRNPA3 antibody reveals that hnRNPA3 selectively binds to sense repeats (repeat probe: S) and antisense repeats (repeat probe: AS), which is inhibited by an excess of the non-biotinylated competitor RNA. **b** Western blotting with an anti-hnRNPA1 antibody reveals that hnRNPA1 binds to sense repeats (repeat probe: S) and antisense repeats (repeat probe: AS), which is inhibited by an excess of the non-biotinylated competitor RNA. C: (A_4_C_2_)_17_-control repeat, S: (G_4_C_2_)_17_-sense repeat, AS: (C_4_G_2_)_17_-antisense repeat

We previously reported that hnRNPA3 binds to the sense repeat RNA and that reduction of hnRNPA3 increases sense repeat RNA foci [41, 42]. We now investigated if hnRNPA3 also binds to the antisense repeat RNA and if reduction of hnRNPA3 increases the number of antisense repeat RNA foci. To verify binding of hnRNPA3 to the antisense repeat RNA, we performed RNA pull down assays using in vitro-transcribed biotinylated RNAs containing either 17 GGGGCC (G_4_C_2_; sense), 17 CCCCGG (C_4_G_2_; antisense), or 17 AAAACC (A_4_C_2_; control) repeats (Suppl. Fig. 1a). Length and amount of the in vitro-transcribed RNA was confirmed by RNA electrophoresis (Suppl. Fig. 1b) and dot blot assays (Suppl. Fig. 1c). Biotinylated in vitro-transcribed RNA probes were incubated with nuclear extracts from HeLa cells in the absence or presence of a 50-fold excess of nonbiotinylated competitor RNA containing the C_4_G_2_ repeat. Bound proteins were eluted using increasing salt concentrations and hnRNPA3 was detected in the eluate by western blotting using an anti-hnRNPA3 polyclonal antibody (Fig. 1a). This elution revealed specific binding of hnRNPA3 not only to the sense but also to the antisense repeat RNA, which was blocked by excess amounts of the non-biotinylated competitor RNA (Fig. 1a). Similarly, the homologous hnRNPA1 [9] also bound specifically to the antisense repeat RNA, and again binding could be significantly reduced by excess amounts of the non-biotinylated competitor RNA (Fig. 1b). Thus, hnRNPA3 interacts with both the sense and the antisense repeat RNA.

### Reduction of hnRNPA3 leads to an increase of antisense RNA foci

**Fig. 2:**
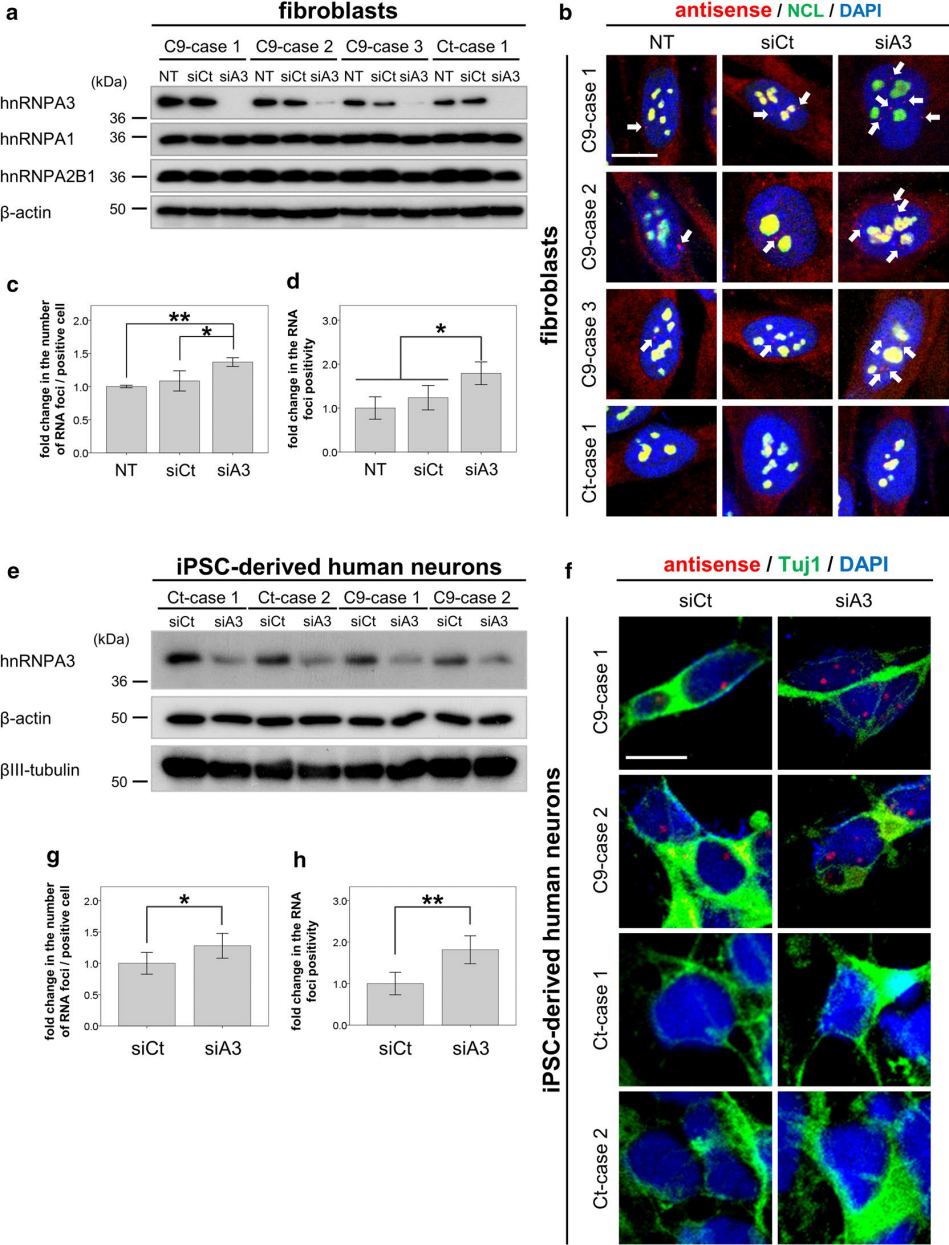
Reduction of hnRNPA3 increases RNA foci number. Knockdown of hnRNPA3 increases antisense RNA foci in fibroblasts derived from *C9orf72* mutation carriers. **a** Western blotting with anti-hnRNPA3, hnRNPA1, and hnRNPA2B1 antibodies confirms selective and efficient siRNA-mediated knockdown of hnRNPA3. β-actin serves as a loading control. **b** Fluorescent in situ hybridization (FISH) of antisense repeat RNA foci in fibroblasts from 3 cases with a *C9orf72* repeat extension (C9) and 1 control case (Ct). Arrows indicate Cy3-positive antisense RNA foci in the nucleus. **c**, **d** Quantification of RNA foci number and positivity upon siRNA-mediated knockdown of hnRNPA3. *N* = 126–234 cells from 3 biological replicates in each line. **e** Western blotting of cell lysates from iPSC-derived human neurons. Western blotting with antibodies to β-actin and βIII-tubulin confirmed selective and efficient knockdown of hnRNPA3. **f** FISH of antisense RNA foci in neurons from control cases (Ct) and *C9orf72* carriers (C9). siCt: control siRNA, siA3: hnRNPA3-targeted siRNA. **g**, **h** Knockdown of hnRNPA3 increases antisense RNA foci number and positivity in iPSC-derived neurons from *C9orf72* mutation carriers. *N* = 72–102 cells from 3 biological replicates in each line. Data of 6 images in each line were used for analysis. *NT* non-treatment, *siCt* control siRNA, *siA3* hnRNPA3-targeted siRNA, *NCL* nucleolin. All graphs are shown as mean ± SEM. *p < 0.05; two tailed paired *t* test. Scale bar 10 μm

To investigate if reduction of hnRNPA3 leads to increased antisense RNA foci as observed before for sense RNA foci [42], we performed fluorescent in situ hybridization (FISH) using three independent fibroblast lines derived from patients with *C9orf72* repeat expansions [42]. Upon siRNA-mediated hnRNPA3 knockdown (Fig. 2a), the number of foci per cell as well as foci positive cell increased compared to control siRNA or mock-treated cells (Fig. 2b–d). Furthermore, in iPSC-derived human neurons generated from fibroblasts derived from C9-case 1 and 2, siRNA-mediated knockdown of hnRNPA3 (Fig. 2e) also led to a significant increase of nuclear antisense RNA foci as well RNA foci positive cell (Fig. 2f–h). Thus, reduced nuclear hnRNPA3 leads to increased formation of sense and antisense repeat RNA foci.

### hnRNPA3 reduction increases DPR generation from the antisense repeat RNA

**Fig. 3:**
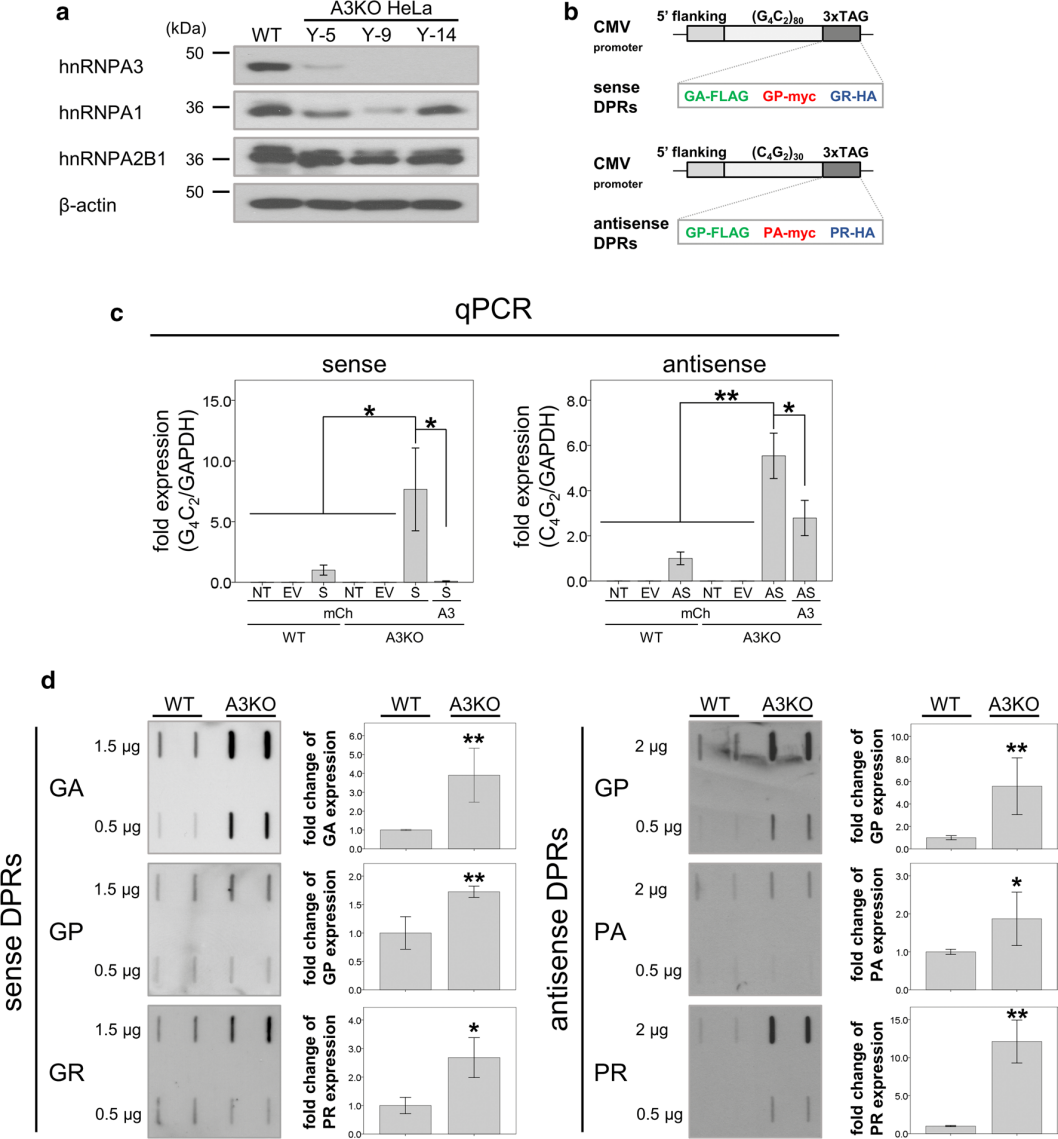
Repeat RNAs and DPRs are increased upon hnRNPA3 knockout. **a** Western blotting confirms selective knockout of hnRNPA3 in cell lines Y-9 and Y-14. Line Y-14 was selected for all following analyses. **b** Schematic representation of cDNA constructs generated to express sense and antisense DPRs fused to individual tags. All graphs are shown as mean ± SEM. *p < 0.05, **p < 0.01; two tailed paired *t* test. **c** Quantification of sense and antisense repeat RNA upon hnRNPA3 knockout and rescue by exogenous expression of hnRNPA3. *N* = 3 biological replicates. *NT* non-treatment, *EV* empty vector, *S* sense repeat RNA, *AS* antisense repeat RNA, *mCh* mCherry, *A3* hnRNPA3, *WT* HeLa wild type cells, *A3KO* HeLa cells with knockout of hnRNPA3. **d** Filter trap assay demonstrating the increase of all DPRs produced from sense and antisense repeat RNA upon hnRNPA3 knockout. *N* = 4 biological replicates. All graphs are shown as mean ± SEM. *p < 0.05, **p < 0.01; two tailed paired t-test

Since knockdown of hnRNPA3 increased not only sense RNA foci [42] but also antisense RNA foci (Fig. 2), we thought that the antisense-derived DPRs poly-PA/PR/GP may also increase concomitantly. To prove this, we first generated hnRNPA3 knockout (A3KO) HeLa cells using Crispr/Cas9 gene editing (Suppl. Fig. 2a, b). We verified the introduction of a frame shift followed by a premature stop codon in selected cell clones (Suppl. Fig. 2b) and then confirmed absence of hnRNPA3 expression by western blotting (Fig. 3a). Clone Y-14 was selected and transfected with sense and antisense repeat constructs that express fluorescent tags in all three reading frames of both repeat encoding DNA strands (Fig. 3b). First, we confirmed by qPCR that repeat RNAs from both strands were increased in the absence of hnRNPA3 (Fig. 3c). Re-expression of hnRNPA3 reduced the increase of repeat RNAs observed in the hnRNPA3 knockout (Fig. 3c). We then used filter trap analyses [31] to quantify DPR production. This revealed that all DPRs made from sense and antisense repeat RNA were significantly increased in the absence of hnRNPA3 (Fig. 3d). Thus, reduction of hnRNPA3 increases sense and antisense repeat RNA foci as well as all DPRs translated from sense and antisense repeat RNA. In contrast, overexpression of hnRNPA3 decreased repeat RNAs (Suppl. Fig. 4a) and DPRs (Suppl. Fig. 4b). These findings indicate that hnRNPA3 is strongly associated with the degradation of repeat RNA and subsequent expression of DPRs.

### hnRNPA3 reduction increases DNA damage in DPR-expressing HeLa cells and C9orf72 patient-derived neurons

**Fig. 4:**
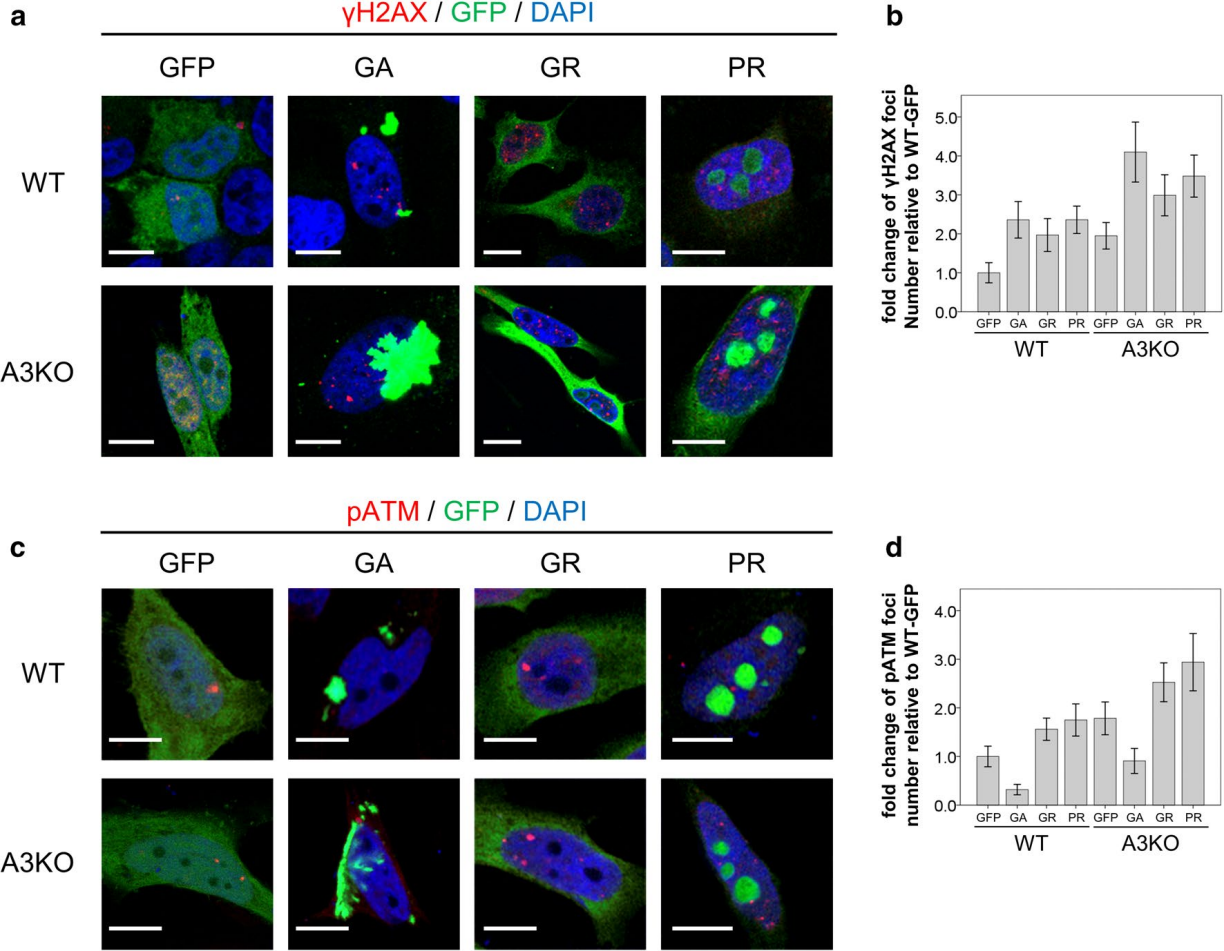
hnRNPA3 knockout enhances DNA damage in DPR expressing HeLa cells. **a** Immunocytochemical detection of γH2AX foci in GFP-tagged DPR-expressing HeLa WT and A3KO HeLa cells. **b** Fold change of γH2AX foci in HeLa WT and A3KO cells expressing the indicated DPRs relative to HeLa WT cells with GFP expression. *N* = 47–127 cells from 2 biological replicates. **c** Immunocytochemical detection of pATM foci in GFP-tagged DPR-expressing HeLa WT and A3KO HeLa cells. **d** Fold change of pATM foci in HeLa WT and A3KO cells expressing the indicated DPRs relative to HeLa WT cells with GFP expression. *N* = 71–263 cells from 2 biological replicates. *GFP* EGFP transfected, *GA* EGFP-tagged poly-GA 175 repeats transfected, *GR* EGFP-tagged poly-GR 177 repeats transfected, PR: EGFP-tagged poly-PR 176 repeats transfected. All graphs are shown as mean ± SEM. *p < 0.05, **p < 0.01; one-way ANOVA and Tukey’s post-hoc test. Scale bar 10 μm

Since reduced hnRNPA3 enhances DSB formation [13], repeat RNA levels, and DPR expression (Figs. 2, 3), we investigated the link between hnRNPA3, DPR expression, and DNA damage/repair. To do so, we determined DPR-dependent DNA damage by quantifying γH2AX and pATM foci in the presence and absence of hnRNPA3. Expression of poly-GA, poly-GR, and poly-PR alone increased γH2AX foci formation in HeLa cells (Fig. 4a, b: *p* < 0.05 or < 0.01, see Suppl. Table 2), which was even more pronounced in the absence of hnRNPA3 (Fig. 4a, b: *p* < 0.05 or < 0.01, see Suppl. Table 2).

Previously, it was reported that cells expressing expanded G_4_C_2_ repeat RNA or poly-GA showed decreased pATM foci in nuclei [58]; however, it is unclear if poly-GR and PR affect pATM. Therefore, we investigated the pATM foci number in cells expressing GFP or GFP-tagged DPR. Interestingly, only poly-GR and poly-PR, but not poly-GA, increased the number of pATM foci in the presence of hnRNPA3 (Fig. 4c, d: *p* < 0.05 or < 0.01, see Suppl. Table 2). Furthermore, in the absence of hnRNPA3, the number of pATM foci were further increased only by poly-GR and poly-PR but not by poly-GA (Fig. 4 c, d: p < 0.05 or < 0.01, see Suppl. Table 2). Surprisingly, even pATM foci formation evoked by GFP expression exceeded the amount of pATM foci evoked by poly-GA (Fig. 4d: *p* < 0.05, see Suppl. Table 2). These findings may suggest that poly-GA inhibits the recruitment of ATM to the site of DNA damage and thus enhances DNA damage by a mechanism different from that of arginine-containing DPRs.

**Fig. 5:**
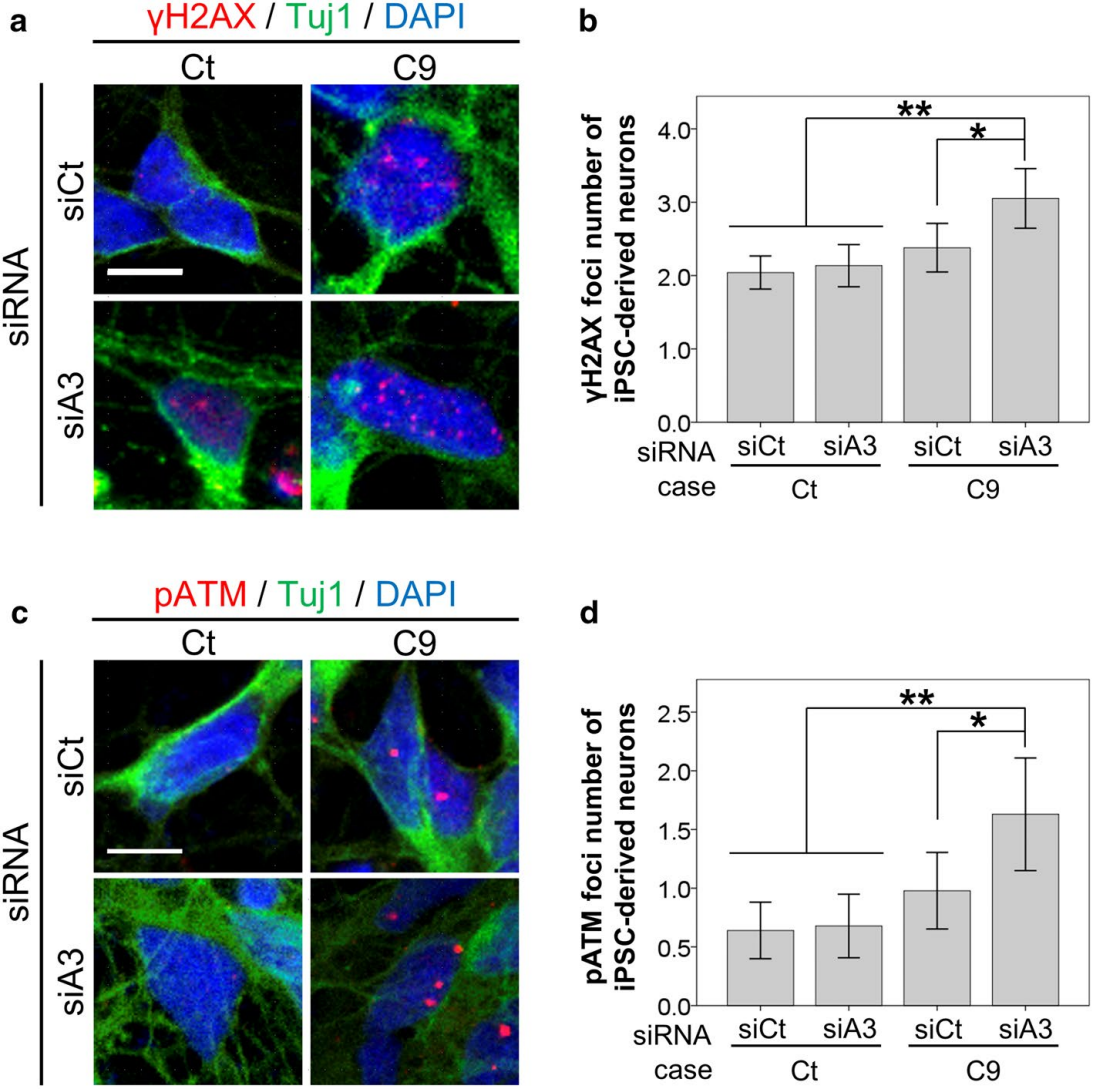
Reduction of hnRNPA3 enhances DNA damage in human neurons. **a** Immunocytochemical detection of γH2AX foci upon siRNA-mediated knockdown of hnRNPA3 in nuclei of motor neurons derived from iPS cells with or without *C9orf72* repeat expansion. **b** Quantification of the number of γH2AX signals observed in **a**. *N* = 721–1558 cells from 2 biological replicates in each line. **c** Immunocytochemical detection of pATM foci upon siRNA-mediated knockdown of hnRNPA3 in the nuclei of neurons derived from iPSCs with or without *C9orf72* repeat expansion. **d** Quantification of the number of pATM signals observed in **c**. *N* = 46–59 cells from 2 biological replicates. *Ct* control case, *C9C9orf72* mutation carriers, *siCt* control siRNA, *siA3* hnRNPA3-targeted siRNA. All graphs are shown as mean ± SEM. *p < 0.05, **p < 0.01; one-way ANOVA and Tukey’s post-hoc test. Scale bar 10 μm

We next asked if increased DNA damage after reduction of hnRNPA3 is also observed in human *C9orf72* patient-derived neurons. siRNA-mediated knockdown of hnRNPA3 significantly increased the number of γH2AX foci in human patient-derived neurons (Fig. 5a, b). Similarly, pATM positive foci (Fig. 5c, d) were also increased upon knockdown of hnRNPA3 in patient-derived human neurons.

### Low hnRNPA3 expression in hippocampal dentate gyri of *C9orf72* patients is associated with enhanced DNA damage

**Fig. 6:**
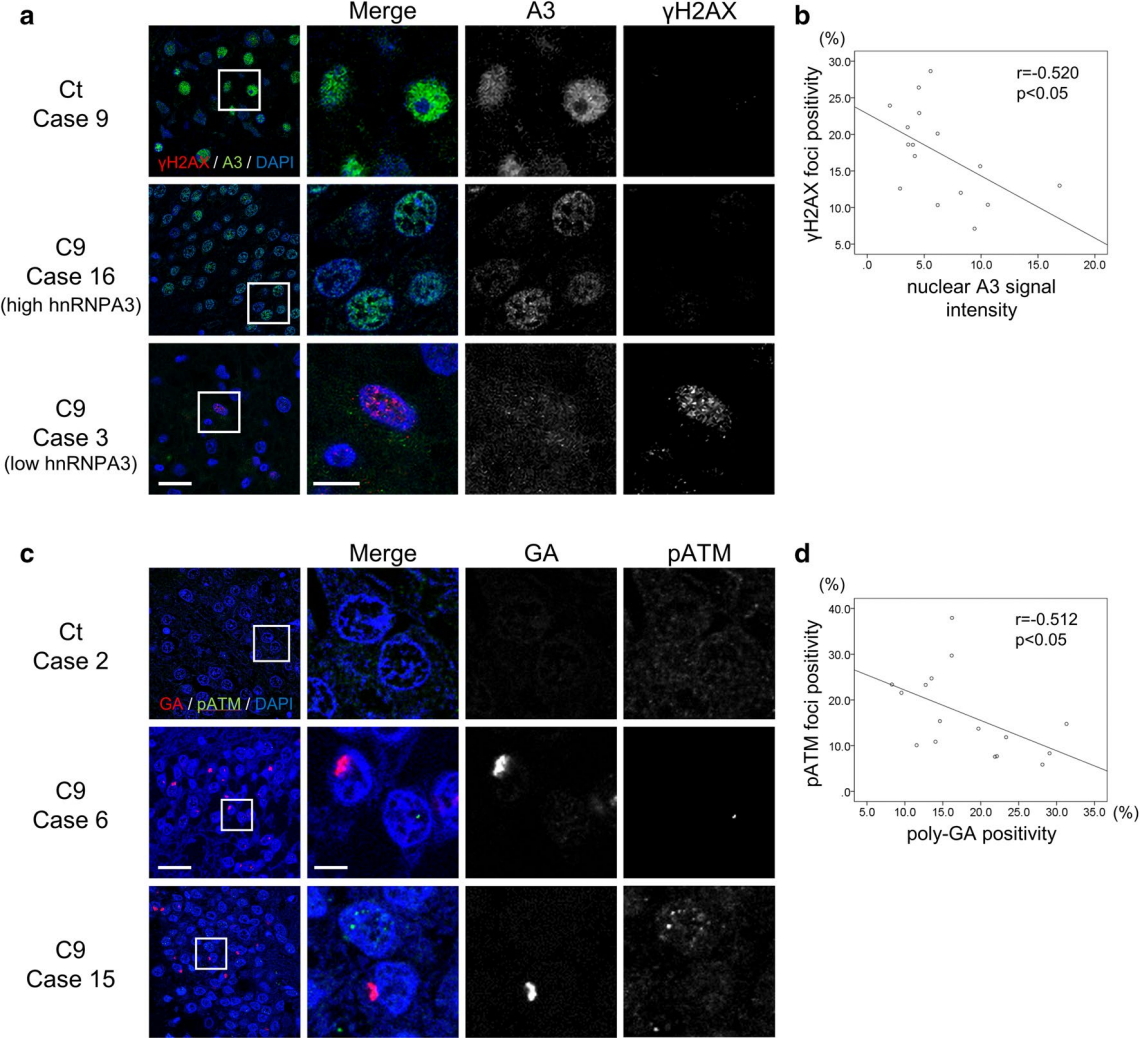
Elevated DNA double strand breaks are associated with reduced hnRNPA3 and pATM foci in the dentate gyrus of *C9orf72* patients. **a** Immunohistochemical detection of hnRNPA3 (A3) and γH2AX foci, a marker for double strand breaks, in granular cells of hippocampal dentate gyri of a control (Ct) case and *C9orf72* carriers (C9) with high (case 16) and low (case 3) nuclear hnRNPA3 expression. Scale bar 25 μm (low magnification figures)/10 μm (high magnification figures). **b** Scatter plot of nuclear hnRNPA3 signal intensities and percentages of nuclei with γH2AX foci of dentate gyrus granular cells in *C9orf72* carriers. Nuclear A3 intensity negatively correlates with γH2AX foci. Pearson’s *r*: − 0.520, *p* < 0.05. *N* = 16 cases, total number of cells counted: 242–636 cells in each case. **c** Immunohistochemical detection of poly-GA and pATM in granular cells of hippocampal dentate gyri of a control (Ct) case and *C9orf72* mutation carriers (C9). Poly-GA positive cells tend not to have nuclear pATM foci. Scale bar 25 μm (low magnification figures)/10 μm (high magnification figures). **d** Scatter plot of percentages of dentate gyrus granular cells with poly-GA inclusions and percentages of granular cells with pATM foci in *C9orf72* carriers. Poly-GA positivity negatively correlates with pATM foci. Pearson’s *r*: − 0.512, *p* < 0.05. *N* = 16 cases, total counted cell number: 185–407 cells in each case

To investigate whether increased DNA damage also occurs in human brains and is dependent on hnRNPA3 expression levels, we measured the correlation between the presence of γH2AX foci, pATM deposits, and poly-GA inclusions with nuclear hnRNPA3 expression in granular cells of the hippocampal dentate gyrus derived from patients with *C9orf72* mutations by immunohistochemistry (Fig. 6a, b, Suppl. Table 3). Indeed, an increased number of γH2AX foci positive cells were found with lowered nuclear signal intensity of hnRNPA3 (Fig. 6a, b; Pearson’s *r*: − 0.520,* p* < 0.05, Suppl. Table 3). Furthermore, the number of granular cells with poly-GA inclusions was negatively correlated with the number of granular cells with pATM foci (Fig. 6c, d. Pearson’s *r*: − 0.512, *p* < 0.05, Suppl. Table 3).

To rule out exacerbated RNA degradation in cases with low hnRNPA3 signals, we investigated the independent RNA binding proteins FUS and HuR (Suppl. Fig. 5). Nuclear signal intensities of both RNA proteins showed no significant correlation with the nuclear signal intensity of hnRNPA3 (Suppl. Fig. 5b, e). Moreover, nuclear signal intensities of FUS and HuR did also not correlate with γH2AX foci positivity (Suppl. Fig. 5c, f).

### Poly-GA sequesters pATM

**Fig. 7:**
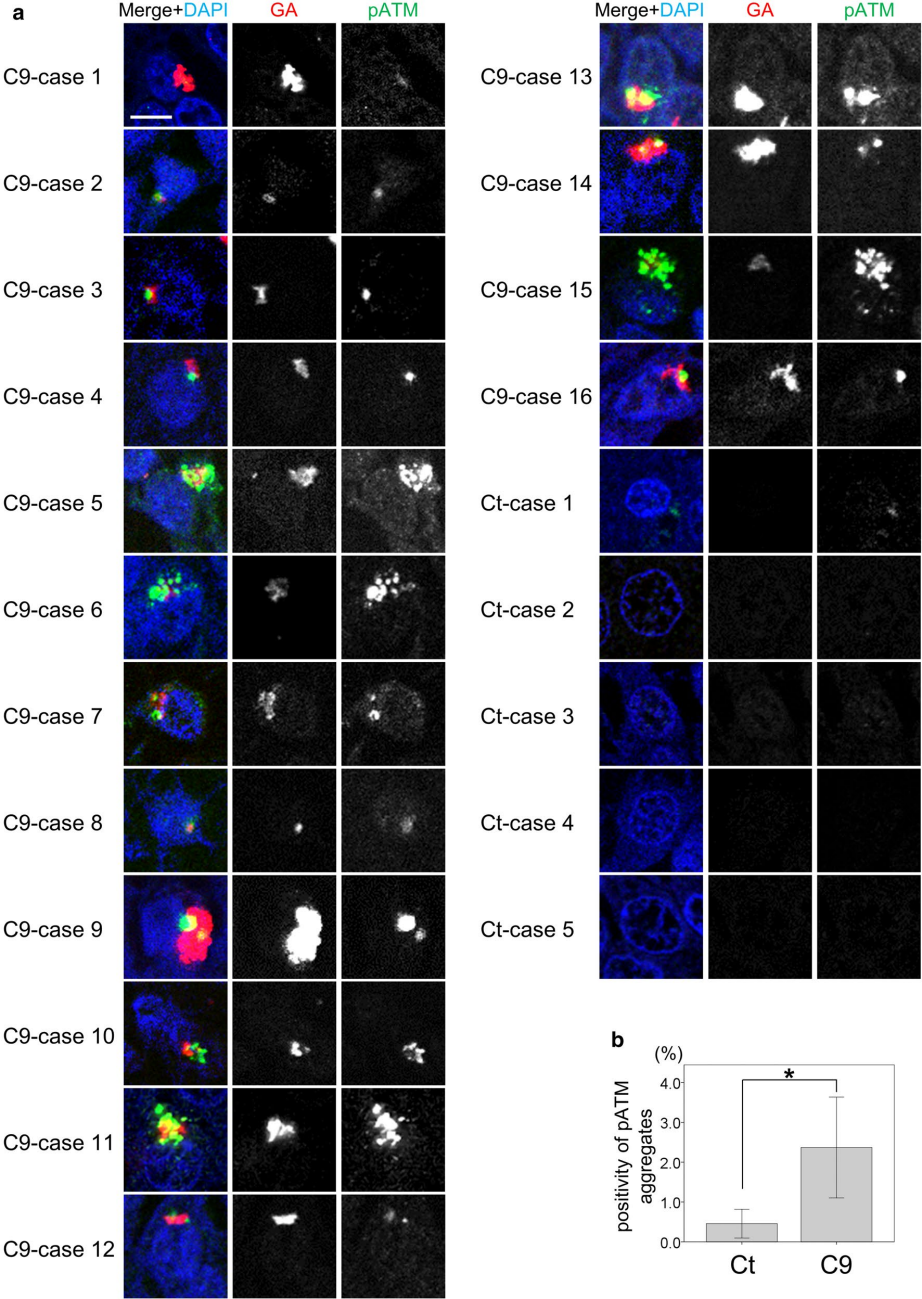
Poly-GA aggregates sequester pATM in cytoplasmic aggregates. **a** Poly-GA aggregates partially colocalize with pATM aggregates in granular cells of hippocampal dentate gyri of *C9orf72* mutation carriers. Scale bar 10 μm. **b** Percentages of dentate gyrus granular cells with cytoplasmic pATM aggregates (diameter > 1 μm) in control cases (*N* = 9) and *C9orf72* mutation carriers (*N* = 16). *p* < 0.05; two tailed paired *t* test. *Ct* control cases, *C9C9orf72* mutation carriers

**Fig. 8:**
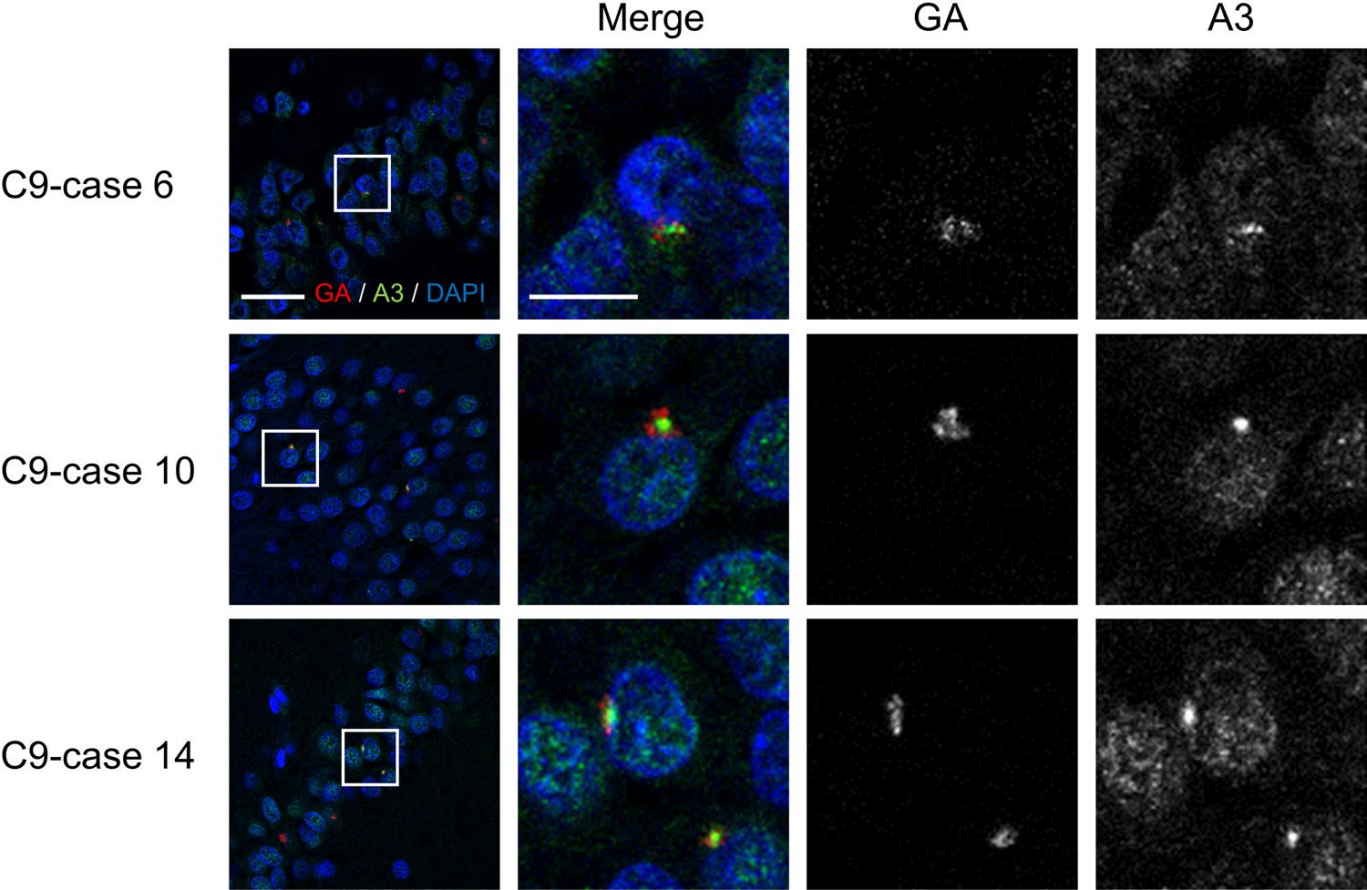
Poly-GA aggregates colocalize with cytoplasmic hnRNPA3 deposits. Immunohistochemical detection of poly-GA (GA) and hnRNPA3 (A3) in granular cells of hippocampal dentate gyri of *C9orf72* carriers (C9). Poly-GA aggregates partially colocalize with cytoplasmic hnRNPA3 deposits in granular cells of hippocampal dentate gyri of *C9orf72* mutation carriers. Scale bar 25 μm (low magnification figures)/10 μm (high magnification figures)

**Fig. 9:**
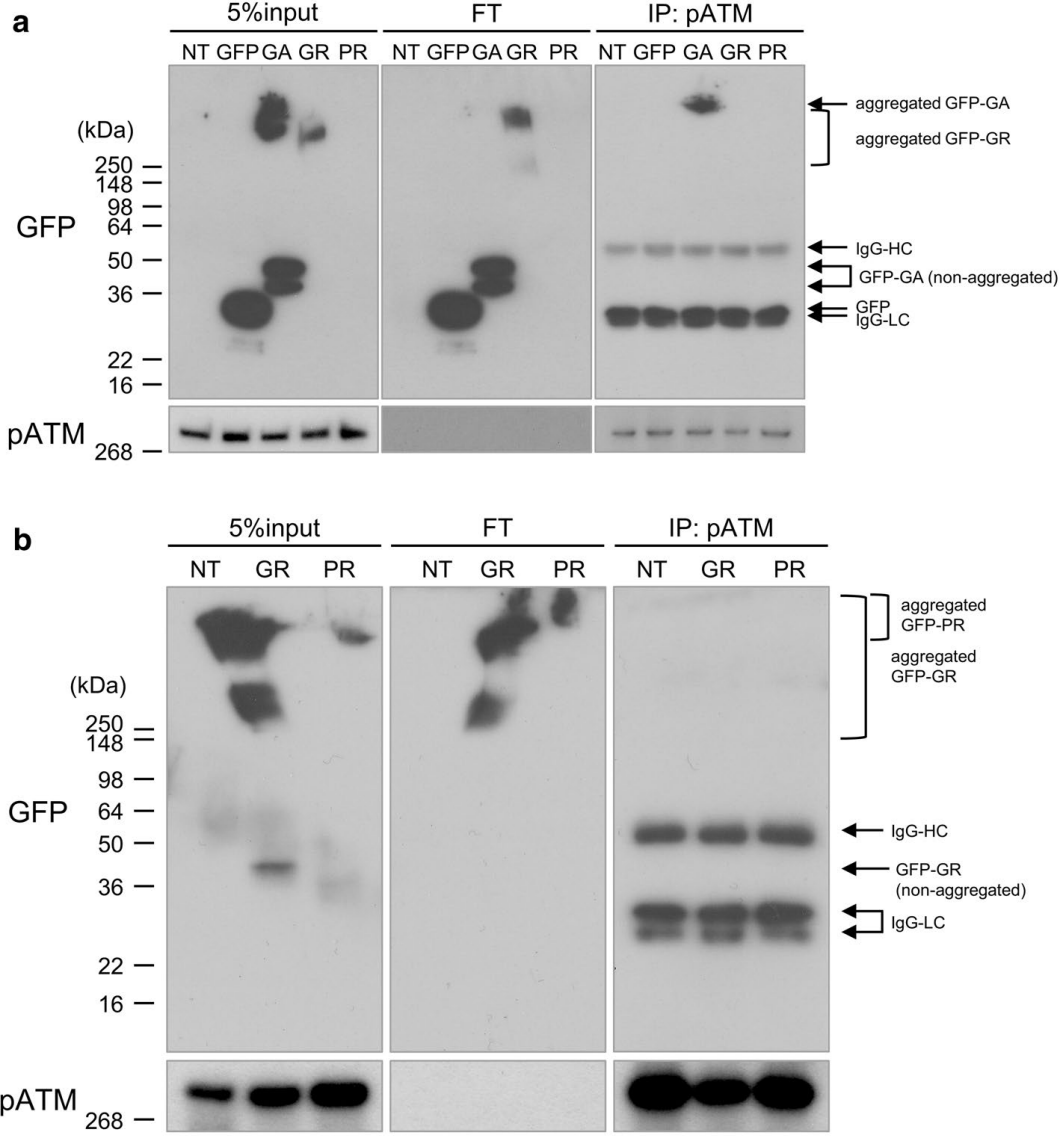
Poly-GA co-immunoprecipitation with pATM. **a** Aggregated poly-GA selectively co-immunoprecipitates with pATM. **b** To increase the sensitivity of DPR detection, cell lysates with 10 × higher protein concentrations as in **a** were used for co-immunoprecipitation. Although aggregated poly-GR and poly-PR are readily detected, only aggregated poly-GA co-immunoprecipitates with pATM. *FT* flow through, *NT* non-transfected, *GFP* EGFP transfected, *GFP-GA/GR/PR* EGFP-tagged poly-GA/GR/PR 89 repeats transfected, *IgG-HC* IgG-heavy chain, *IgG-LC* IgG-light chain

Notably, all C9 cases contained cytoplasmic pATM aggregates partially colocalizing with poly-GA inclusions (Fig. 7a, Suppl. Fig. 6a). On the other hand, control cases only rarely contained cytoplasmic pATM deposits, which were additionally much smaller than those in C9 cases (Fig. 7a, b, Suppl. Fig. 6b). Interestingly, hnRNPA3 aggregates also colocalize with poly-GA inclusions (Fig. 8), which is consistent with our previous study [42]. Therefore, we hypothesized that poly-GA aggregation sequesters pATM in cytoplasmic aggregates. To confirm co-aggregation of poly-GA and pATM, we performed co-immunoprecipitations in GFP-GA transfected HeLa cells (Fig. 9a). Strikingly, the anti-pATM antibody selectively co-immunoprecipitated aggregated poly-GA, which accumulated in the stacker of the gel during gel electrophoresis (Fig. 9a). In contrast, non-aggregated poly-GA was not precipitated (Fig. 9a). Poly-GR and poly-PR were not co-precipitated by the anti-pATM antibody even when using cell lysates with higher amounts of both DPRs for the co-immunoprecipitation assays (Fig. 9b; compare to Fig. 9a). Furthermore, the anti-pATM antibody did not cross-react with aggregated poly-GA (Suppl. Fig. 7) demonstrating the specificity of the co-immunoprecipitation in Fig. 9a. To further confirm that poly-GA aggregates sequester pATM in the cytoplasm, where most GA deposits occur [43], we performed co-immunoprecipitations upon from nuclear and cytoplasmic fractions (Suppl. Fig. 8a). Aggregated poly-GA selectively co-precipitated with pATM in the cytoplasmic fraction (Suppl. Fig. 8b), while aggregated poly-GA was not detected in the nuclear fraction and consequently no co-immunoprecipitation was observed (Suppl. Fig. 8c). Poly-GR and poly-PR did not co-precipitate with pATM in the nuclear fractions or the cytoplasmic fractions (Suppl. Fig. 8b–d). Thus, we conclude that poly-GA deposits selectively sequester pATM within the cytoplasm.

## Discussion

Our study shows the functional and pathological consequences of loss of hnRNPA3 observed in *C9orf72*-associated neurodegeneration. First, hnRNPA3 not only binds to sense RNA repeats but also to antisense RNA repeats (Fig. 1). Second, lowering hnRNPA3 increases sense and antisense repeat RNAs, leading to enhanced RNA foci production as well as increased DPR generation (Figs. 2, 3). Third, reduced hnRNPA3 enhances DSB formation, which is induced by DPR (Fig. 4) and likely also by RNA foci (Fig. 5). Finally, poly-GA aggregates but no other DPRs sequester pATM within the cytoplasm and impede the recruitment of pATM to sites of DNA damage (Figs. 6, 7, 9, Suppl. Figs. 6, 8). Thus, the loss of hnRNPA3 expression likely potentiates DNA damage in *C9orf72* patients’ brains.

In RNA pull-down assays we demonstrate that antisense repeat RNAs bind to hnRNPA3 similarly to sense repeat RNAs (Fig. 1a). Likewise, hnRNPA1 binds to both repeat transcripts (Fig. 1b). hnRNPA1 and A3 both share very similar RNA recognition motifs (RRM) [1, 59]. However, despite similar binding efficiencies, reduced hnRNPA3 increases poly-GA expression more than reduced hnRNPA1 [42].

Reduced nuclear hnRNPA3 expression positivity correlates with higher γH2AX foci in dentate gyrus granular cells of patients with *C9orf72* mutations (Fig. 6a, b). This is in line with our cell culture models using HeLa cells and iPSC-derived human neurons (Figs. 4, 5). These findings indicate that reduction of hnRNPA3 further promotes DSB via increased RNA foci and DPR expression. This is also consistent with the finding that reduction of hnRNPA3 itself independently promotes DSB [13]. Indeed, depletion of hnRNPA3 increased γH2AX and pATM foci even in the cells with GFP only expression (Fig. 4). Thus, reduction of hnRNPA3 alone may enhance DSB, which is further increased by RNA foci and DPR expression.

Nuclear pATM foci as well as γH2AX foci are increased upon reduction of hnRNPA3 in cells expressing poly-GR and poly-PR (Fig. 4). In contrast, poly-GA decreased nuclear pATM foci, and, consequently, poly-GA deposition negatively correlated with nuclear pATM foci (Figs. 4c, d, 6c, d). Interestingly, cytoplasmic pATM aggregates were found in the dentate gyri of *C9orf72* patients but only rarely in control cases (Fig. 7a, b). Co-IPs revealed that pATM bound selectively to aggregated poly-GA but not to soluble poly-GA (Fig. 9, Suppl. Fig. 8), which is in line with the partial co-localization of poly-GA deposits with pATM (Fig. 7a). These findings, therefore, suggest that pATM is selectively sequestered by poly-GA deposits and that sequestration impairs DSB repair in *C9orf72* cases. Interestingly, it was reported that mutant huntingtin also sequesters pATM in the cytoplasm and impairs the recruitment of pATM to sites of irradiation-induced DSB [16]. Therefore, we speculate that pATM sequestration might be a common feature in repeat expansion disorders. However, alternative pathways such as defective nucleo-cytoplasmic transport may also impair pATM recruitment. ATM exists as a dimer in the cytoplasm [3, 56]. In response to DSB, ATM is oxidized, monomerized, and auto-phosphorylated. Subsequently, pATM binds to importin and shuttles into the nucleus [56]. DPR and *C9orf72* repeat RNA are thought to impair nucleocytoplasmic transport [19, 28, 65, 66]. Therefore, nucleocytoplasmic transport dysfunction may additionally impair the recruitment of pATM to the nucleus. Walker et al. reported that repeat RNA increased γH2AX foci, and decreased pATM foci in the nucleus [58]. They concluded that activation of ATM was impaired by DPRs and repeat RNA. We now demonstrate that DPRs may affect ATM activation via differential individual mechanisms, i.e., poly-GA, but not poly-GR or poly-PR, sequesters ATM in cytoplasmic deposits. We have not further investigated how poly-GR and poly-PR enhance DSB without sequestration of pATM; however, there are some reports that show an association of poly-GR and poly-PR with DNA damage repair. Lopez-Gonzalez et al. reported that poly-GR binds to mitochondrial ribosomal proteins and leads to mitochondrial dysfunction, oxidative stress, and DNA damage [32]. Others reported that poly-GR and poly-PR change the intranuclear distribution of NPM1, which is protective against DNA damage [15, 54, 62]. Thus, each neurotoxic DPR may affect DNA damage in an individual way. On the other hand, the prevalence of poly-GR and poly-PR depositions is much lower than poly-GA deposits in human brains [51]. Poly-GA may, therefore, be the most relevant DPR in the pathological cascade of DNA damage in human brains.

Finally, we have not fully defined how H2AX is phosphorylated in the absence of pATM recruitment. After pATM is recruited to sites of DSB, pATM normally phosphorylates H2AX and converts it to γH2AX [10]. Thus, it is rather surprising that poly-GA increases γH2AX while decreasing pATM. We speculate that under these conditions γH2AX is phosphorylated by the poly-GA induced DNA-PK, which is a known H2AX kinase [53]. Therefore, DNA-PK may be able to phosphorylate H2AX at the sites of DSB, though precipitation of pATM into poly-GA aggregates may nevertheless cause a loss of function.

**Fig. 10:**
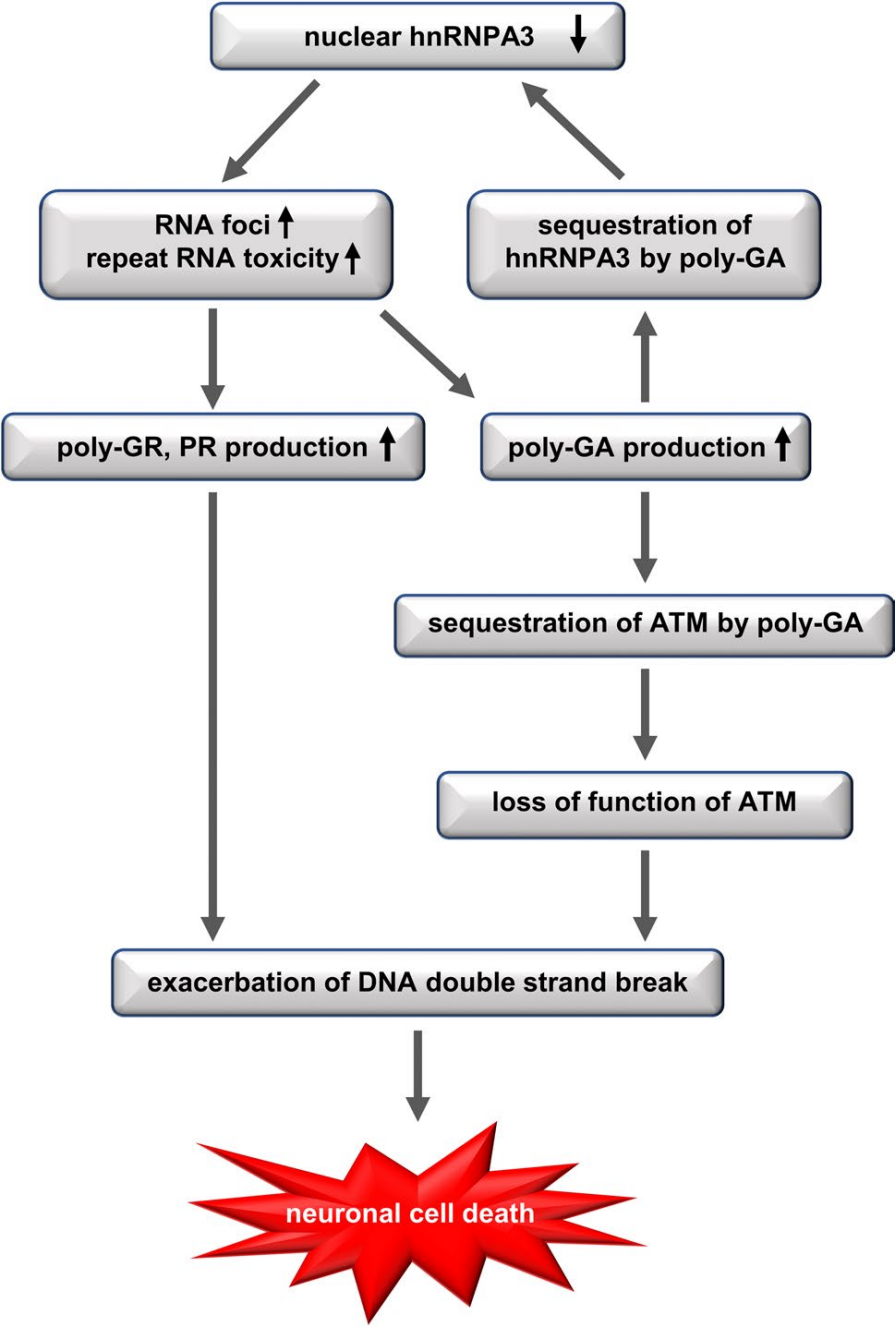
Schematic model demonstrating the cellular consequences of reduced hnRNPA3

Taken together, we conclude that reduced hnRNPA3 enhances DNA damage (1) by increasing (G_4_C_2_)*n* and (C_4_G_2_)*n* expressions and foci formation (2) by increasing DPR expression, and (3) by impairment of ATM-mediated DNA damage repair through the sequestration of pATM by poly-GA (Fig. 10).

## Electronic supplementary material

Below is the link to the electronic supplementary material.

Supplementary file1 (PDF 20669 kb)
