# Supplementary material for: Poly-glycine–alanine exacerbates C9orf72 repeat expansion-mediated DNA damage via sequestration of phosphorylated ATM and loss of nuclear hnRNPA3
Source: Acta Neuropathol. 2019 Oct 23;139(1):99–118. doi: 10.1007/s00401-019-02082-0 (PMC6942035; doi:10.1007/s00401-019-02082-0)
Supplement: Supplementary file 1 — Supplementary file1 (PDF 20669 kb) [file 401_2019_2082_MOESM1_ESM.pdf]

Supplementary Figures

Poly-Glycine-Alanine exacerbates *C9orf72* repeat expansion-mediated DNA damage via sequestration of phosphorylated ATM and loss of nuclear hnRNPA3

Yoshihiro Nihei[1], Kohji Mori[2], Georg Werner[3], Thomas Arzberger[1, 4, 5], Qihui Zhou[1], Barham Khosravi[1], Julia Japtok[6], Andreas Hermann[7, 8], Andreas Sommacal[9], Markus Weber[10], German Consortium for Frontotemporal Lobar Degeneration, Bavarian Brain Banking Alliance, Frits Kamp[3], Brigitte Nuscher[3], Dieter Edbauer[1, 11] and Christian Haass[1, 3, 11]

[1] German Center for Neurodegenerative Diseases (DZNE) Munich, 81377 Munich, Germany; [2] Department of Psychiatry, Osaka University Graduate School of Medicine, Yamadaoka 2-2 D3, Suita, Osaka 565-0871, Japan; [3] Metabolic Biochemistry, Biomedical Center (BMC), Faculty of Medicine, Ludwig-Maximilians-Universität München, 81377 Munich, Germany; [4] Center for Neuropathology and Prion Research, Ludwig-Maximilians-Universität München, 81377 Munich, Germany; [5] Department of Psychiatry and Psychotherapy, Ludwig-Maximilians Universität München, 80336 Munich, Germany; [6] Department of Neurology, Technische Universität Dresden, 01307 Dresden, Germany; [7] Translational Neurodegeneration Section „Albrecht-Kossel“, Department of Neurology and Center for Transdisciplinary Neurosciences Rostock (CTNR), University Medical Center Rostock, University of Rostock, 18147 Rostock, Germany; [8] German Center for Neurodegenerative Diseases (DZNE) Rostock/Greifswald, 18147 Rostock, Germany; [9] Institute for Pathology, Kantonsspital St. Gallen, Rorschacher Strasse 95, CH-9007 St. Gallen, Switzerland; [10] Muskelzentrum/ALS Clinic, Kantonsspital St. Gallen, Rorschacher Strasse 95, CH-9007 St. Gallen, Switzerland; [11] Munich Cluster for System Neurology (SyNergy), 81377 Munich, Germany.

To whom correspondence should be addressed:
Christian Haass
e-mail: christian.haass@mail03.med.uni-muenchen.de

Supplementary Figure Legends

Suppl. Fig. 1 Schematic presentation of repeat RNAs used in the pull-down assays
a Sequence of the control, sense, and antisense RNA. AC17: control, GC17: sense repeat, CG17: antisense repeat. Restriction sites of NheI / HindIII are underlined. **b** RNA electrophoresis of in vitro transcribed sense, antisense, and control repeat RNAs. CG17-competitor: non-biotinylated antisense repeat. **c** Dot blot assay to confirm efficacy of biotinylation.

Suppl. Fig. 2 CRISPR/Cas9-mediated hnRNPA3 knockout (A3KO) in HeLa cells
a Strategy to target the human hnRNPA3 locus indicating the target sequence (red) and the protospacer adjacent region (PAM, black). **b** Sequence confirmation of a single base deletion in cell line Y14, which leads to a frame shift and an early stop codon. asterisk: stop codon.

Suppl. Fig. 3 Areas of hippocampal dentate gyrus analyzed by immunohistochemistry
Hematoxylin-eosin stain of the human dentate gyrus with adjacent cornu ammonis regions.
Frames represent the three areas selected for images of granular cells in each case. Scale bar = 1mm. CA3: cornu ammonis region 3, CA4: cornu ammonis region 4, gl: granular layer of dentate gyrus, ml: molecular layer of dentate gyrus, pl: polymorphic layer of dentate gyrus

Suppl. Fig. 4 Decrease of repeat RNAs and DPRs upon hnRNPA3 overexpression
a Quantification of sense and antisense repeat RNA upon exogeneous hnRNPA3 expression. N=3 biological replicates. **b** Filter trap assay demonstrating the increase of all DPRs produced from sense and antisense repeat RNA upon hnRNPA3 knockout. N=6 biological replicates. NT: non-treatment, EV: empty vector, S: sense repeat RNA, AS: antisense repeat RNA, mCh: mCherry, mCh-A3: mCherry-tagged hnRNPA3. All graphs are shown as mean ± SEM. *p<0.05, **p<0.01; two tailed paired t-test.

Suppl. Fig. 5 Signal intensity of either nuclear FUS or HuR is not associated with nuclear hnRNPA3 or γH2AX foci positivity.
a Immunohistochemical detection of hnRNPA3 (A3) and FUS in granular cells of hippocampal dentate gyri of C9orf72 carriers (C9). Scale bar: 10 µm. **b** Nuclear A3 intensity does not correlate with FUS. Scatter plot of nuclear A3 and FUS signal intensities of dentate gyrus granular cells in C9orf72 carriers. Pearson's r=0.298, p =0.280. **c** Nuclear FUS intensity does not correlate with γH2AX foci. Scatter plot of nuclear FUS and percentages of nuclei with γH2AX foci of dentate gyrus granular cells in C9orf72 carriers. Pearson's r=0.016, p =0.995. N=15 cases, total number of cells counted: 132-645 cells in each case. **d** Immunohistochemical detection of nuclear A3 and HuR in granular cells of hippocampal dentate gyri of C9orf72 carriers (C9). Scale bar: 10 µm. **e** Nuclear A3 intensity does not correlate with HuR. Scatter plot of nuclear A3 and HuR signal intensities of dentate gyrus granular cells in C9orf72 carriers. Nuclear A3 intensity does not correlate with HuR. Pearson's r=0.279, p =0.314. **f** Nuclear HuR intensity does not correlate with γH2AX foci. Scatter plot of nuclear HuR and percentages of nuclei with γH2AX foci of dentate gyrus granular cells in C9orf72 carriers. Nuclear HuR intensity does not correlate with γH2AX foci. Pearson's r=0.040, p =0.888. N=15 cases, total number of cells counted: 177-675 cells in each case.

Suppl. Fig. 6 Granular cells of dentate gyri co-stained with anti-pATM and poly-GA antibodies
a Low magnification figures of granular cell layers of dentate gyri from C9orf72 mutation carrier (C9-case 11) and a control case (Ct-case 1). C9orf72 mutation carriers generally have larger pATM aggregates in granular cell layers than in control cases. pATM aggregates in C9orf72 mutation carriers show partial colocalization with poly-GA aggregates. Square parts were shown in Fig. 7 as high magnification figures. **b** Granular cells of dentate gyri from control cases (Ct-case 6-9). Cytoplasmic pATM aggregates are less frequent and smaller in control cases than in C9orf72 mutation carriers.

Suppl. Fig. 7 Anti-pATM antibody does not cross-react with aggregated poly-GA
Immunodetection of GFP-tagged DPRs overexpressed HeLa cells and endogenous pATM with an anti-pATM antibody (left) and anti-GFP antibody (right). Proteins were separated on 3-8% gradient Tris-Acetate gels and samples were transferred to PVDF membrane with 25V, 15 hours. Note that the anti-GFP antibody detects a smear of aggregated poly-GA, whereas the anti-pATM antibody detects a discrete band of approximately 370 kDa (arrow).

Suppl. Fig. 8 Aggregated GFP-tagged poly-GA co-precipitates with pATM in cytoplasmic fractions only
a Subcellular fractionation was confirmed by immunoblotting for cytoplasmic (α-tubulin) and nuclear (Lamin A/C) markers. **b** Selective co-immunoprecipitation of aggregated GFP-GA with pATM from cytoplasmic fractions. GFP-PR was not detected in this assay. **c** GFP-tagged DPRs including poly-GA do not co-immunoprecipitate with pATM from nuclear fractions. **d** Co-immunoprecipitations using 5-fold concentrated cytoplasmic and nuclear fractions for the detection of GFP-GR and GFP-PR still did not allow the detection of GFP-GR or GFP-PR co-precipitated with pATM.

a

control AAAACC(A_4C_2)17 repeats, 124 base

: GGGAGACCCAAGCUGGCUAGCAAAACCAAAACCAAAACCAAAACCAA
AACCAAAACCAAAACCAAAACCAAAACCAAAACCAAAACCAAAACCA
AAACCAAAACCAAAACCAAAACCAAAACCA

sense: GGGGCC(G_4C_2)17 repeats, 124 base

GGGAGACCCAAGCUGGCUAGCGGGGCCGGGGCCGGGGCCGGGGCCGG
GGCCGGGGCCGGGGCCGGGGCCGGGGCCGGGGCCGGGGCCGGGGCCG
GGGCCGGGGCCGGGGCCGGGGCCGGGGCCA

antisense: CCCCGG(C_4G_2)17 repeats, 124 base

GGGAGACCCAAGCUGGCUAGCCCCCGGCCCCGGCCCCGGCCCCGGCC
CCGGCCCCGGCCCCGGCCCCGGCCCCGGCCCCGGCCCCGGCCCCGGC
CCCGGCCCCGGCCCCGGCCCCGGCCCCGGA

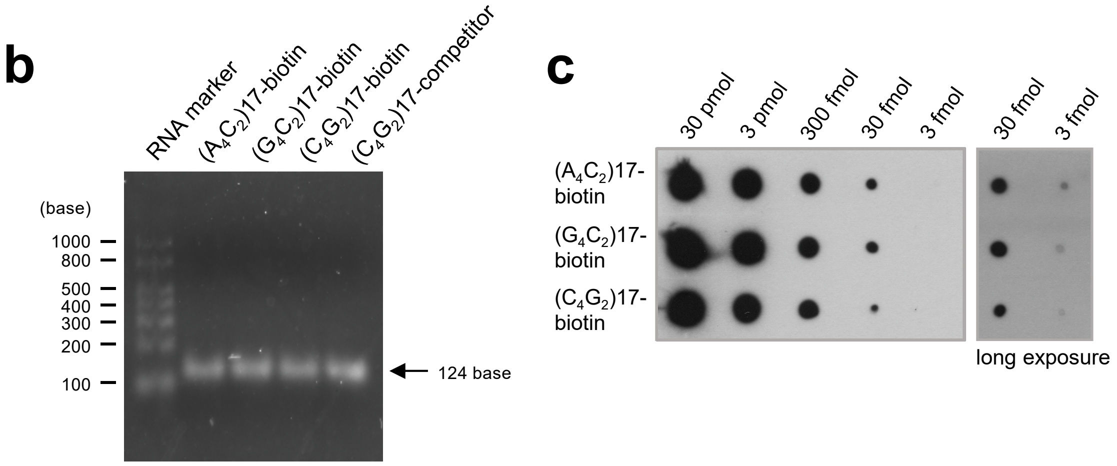

Suppl. Fig. 1

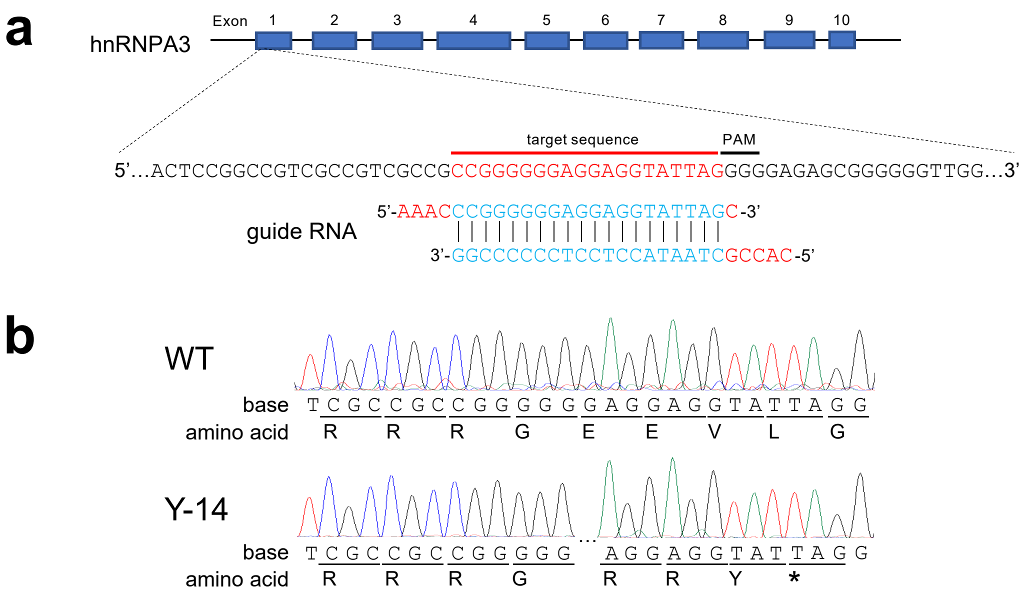
a
hnRNPA3
Exon 1 2 3 4 5 6 7 8 9 10
target sequence
PAM
5'…ACTCCGGCCGTCGCCGTCGCCGCCGGGGGGAGGAGGTATTAGGGGGAGAGCGGGGGGTTGG…3'
guide RNA
5'-AAACCCGGGGGGAGGAGGTATTAGC-3'
3'-GGCCCCCCTCCTCCATAATCGCCAC-5'
b
WT
base TCGCCGCCGGGGGAGGAGGTATTAGG
amino acid R R R G E E V L G
Y-14
base TCGCCGCCGGGGG…AGGAGGTATTAGG
amino acid R R R G R R Y *

Suppl. Fig. 2

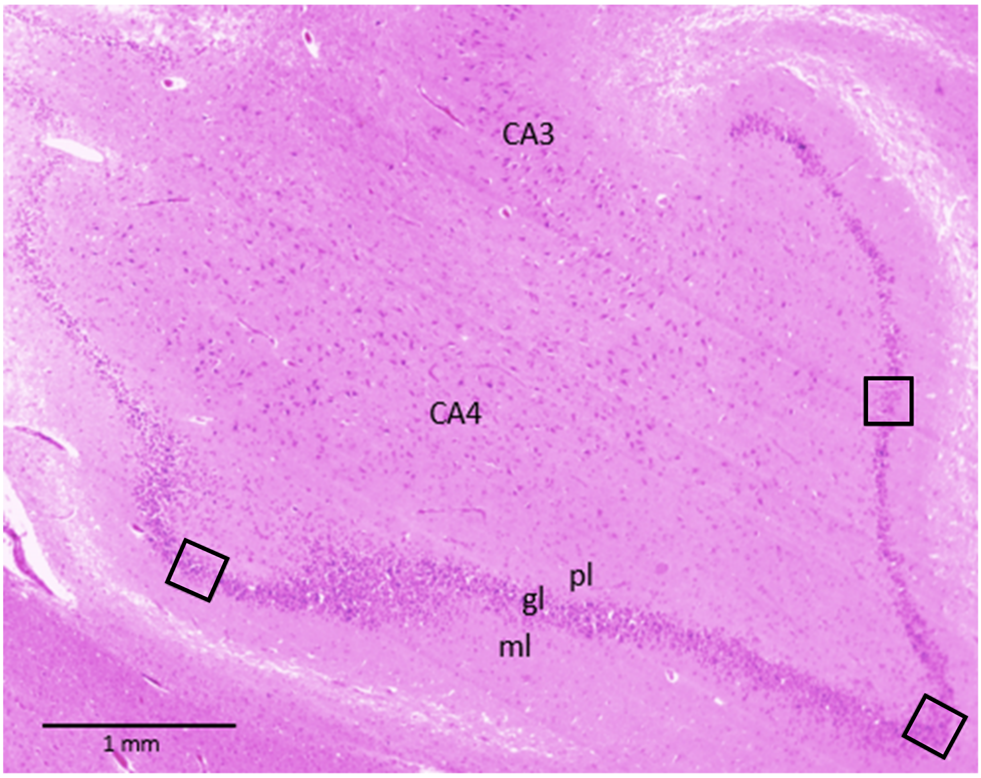

Suppl. Fig. 3

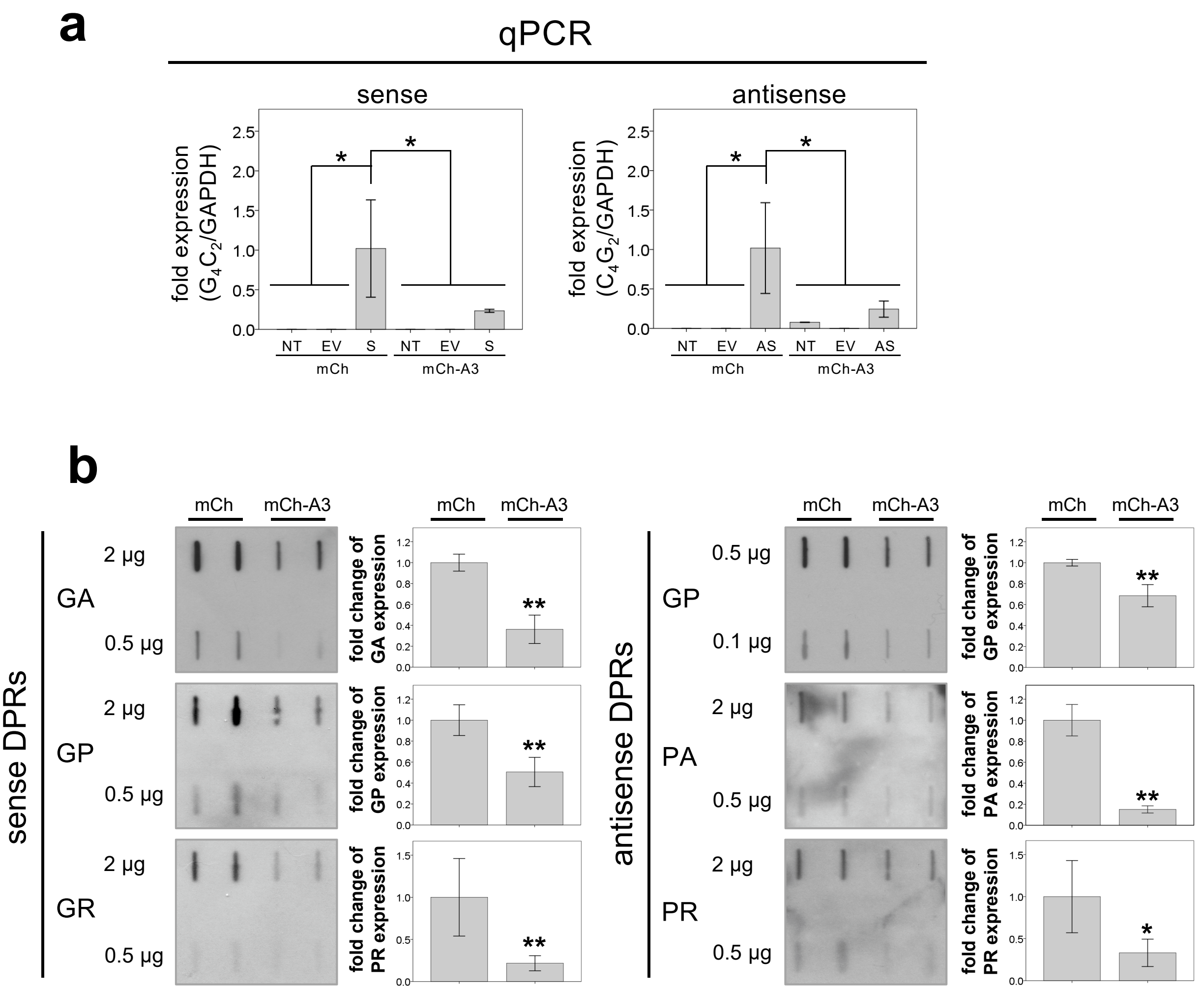
a
qPCR
sense
antisense
fold expression (G4C2/GAPDH)
fold expression (C4G2/GAPDH)
NT EV S NT EV S
NT EV AS NT EV AS
mCh
mCh-A3
b
sense DPRs
antisense DPRs
GA
GP
GR
PA
PR
2 µg
0.5 µg
0.1 µg
fold change of GA expression
fold change of GP expression
fold change of PR expression
fold change of PA expression

Suppl.Fig. 4

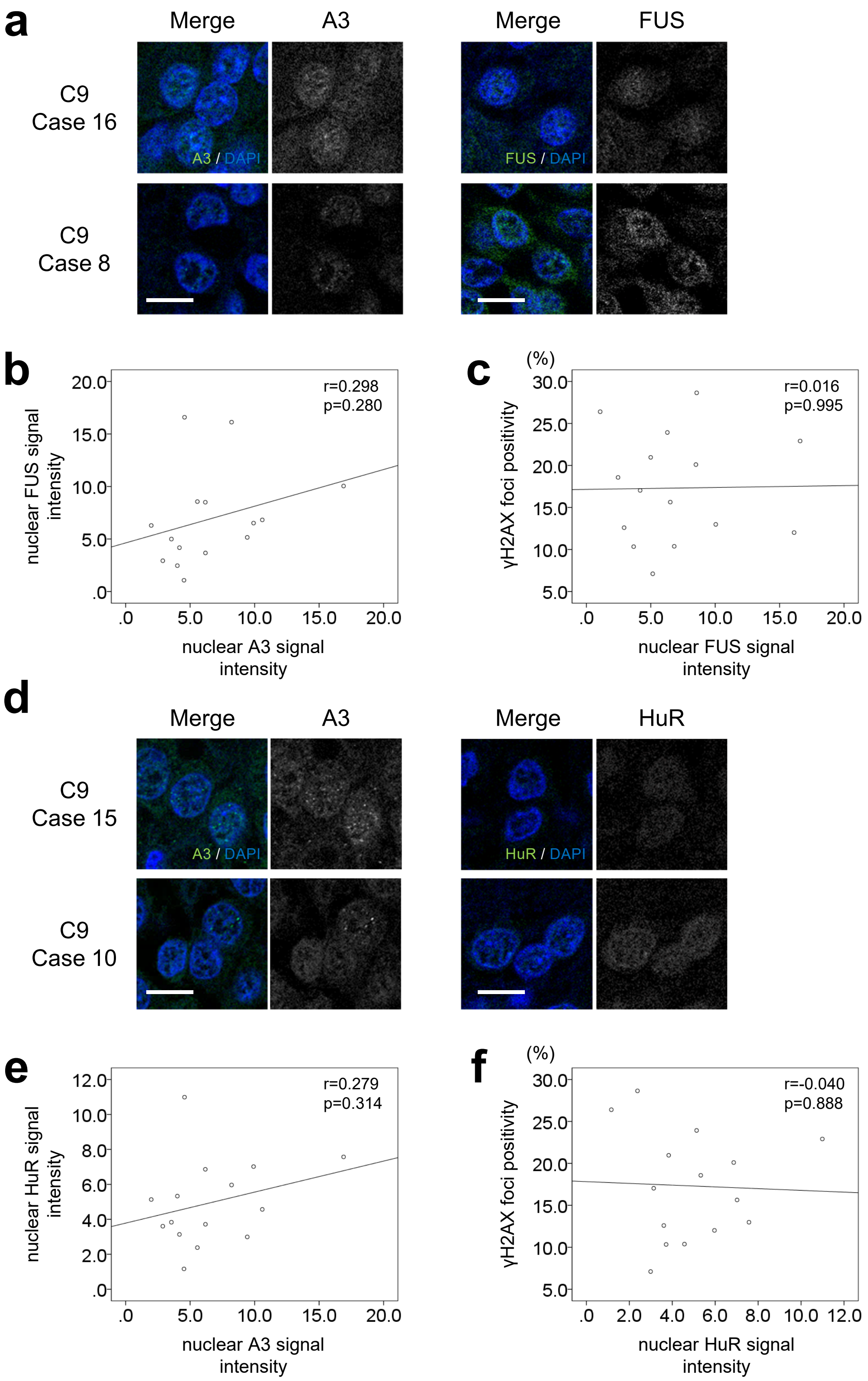
a
Merge
A3
Merge
FUS
C9
Case 16
A3 / DAPI
FUS / DAPI
C9
Case 8
b
nuclear FUS signal intensity
20.0
15.0
10.0
5.0
.0
r=0.298
p=0.280
.0
5.0
10.0
15.0
20.0
nuclear A3 signal intensity
c
(%)
γH2AX foci positivity
30.0
25.0
20.0
15.0
10.0
5.0
r=0.016
p=0.995
.0
5.0
10.0
15.0
20.0
nuclear FUS signal intensity
d
Merge
A3
Merge
HuR
C9
Case 15
A3 / DAPI
HuR / DAPI
C9
Case 10
e
nuclear HuR signal intensity
12.0
10.0
8.0
6.0
4.0
2.0
.0
r=0.279
p=0.314
.0
5.0
10.0
15.0
20.0
nuclear A3 signal intensity
f
(%)
γH2AX foci positivity
30.0
25.0
20.0
15.0
10.0
5.0
r=-0.040
p=0.888
.0
2.0
4.0
6.0
8.0
10.0
12.0
nuclear HuR signal intensity

Suppl. Fig. 5

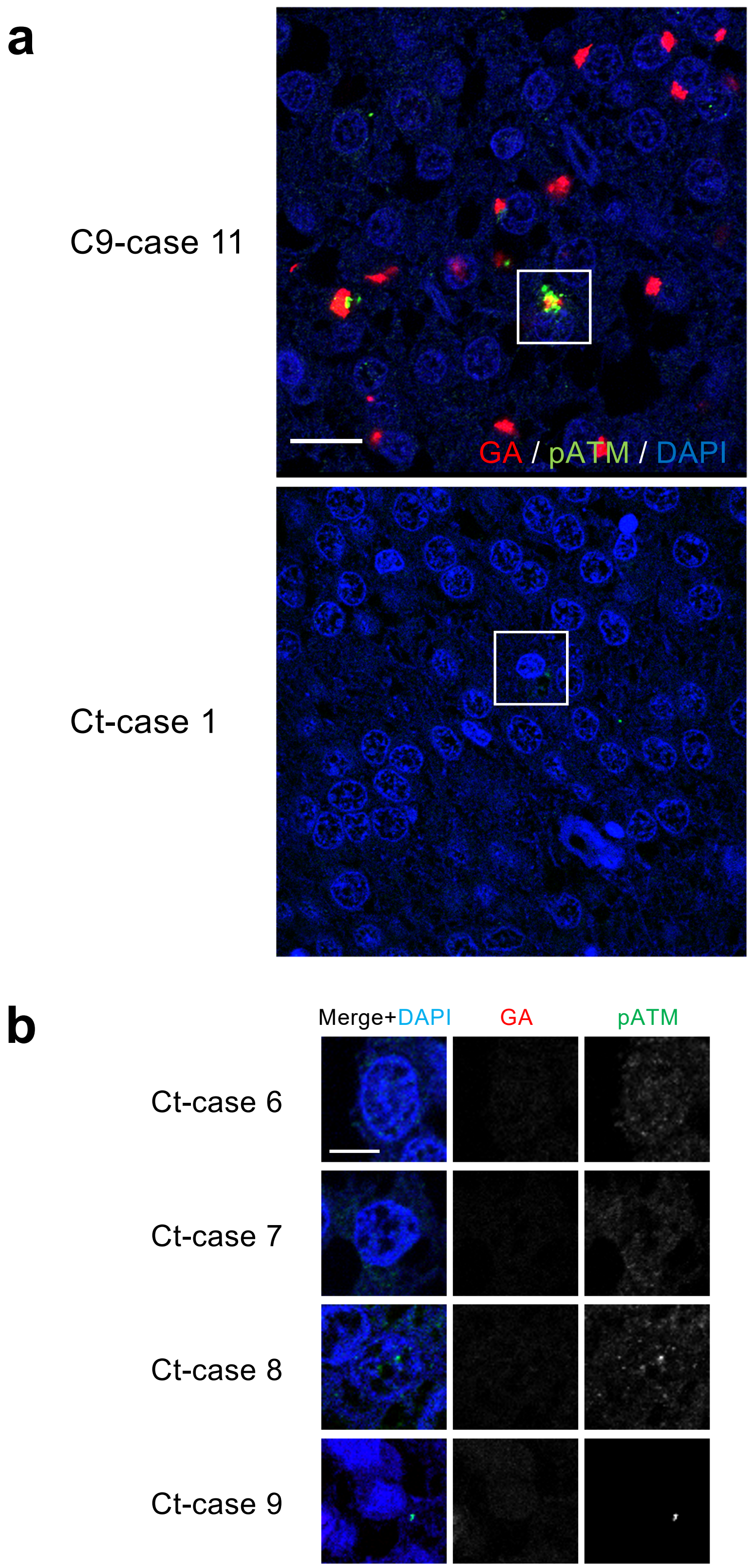
a
C9-case 11
GA / pATM / DAPI
Ct-case 1
b
Merge+DAPI
GA
pATM
Ct-case 6
Ct-case 7
Ct-case 8
Ct-case 9

Suppl. Fig. 6

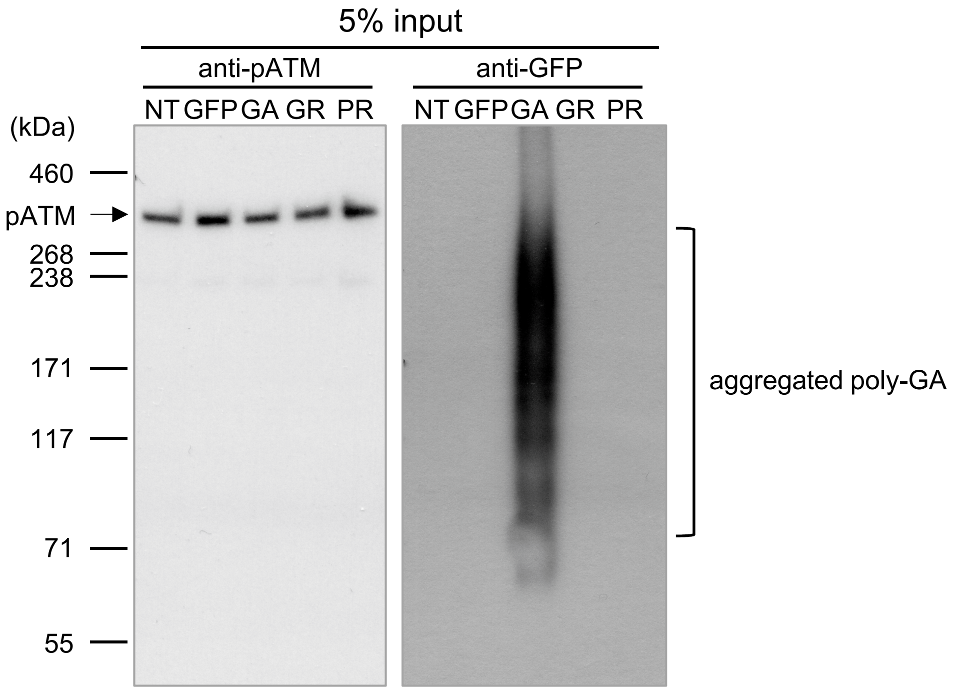
5% input
anti-pATM
anti-GFP
NT GFP GA GR PR
NT GFP GA GR PR
(kDa)
460
pATM
268
238
171
117
71
55
aggregated poly-GA

Suppl. Fig. 7

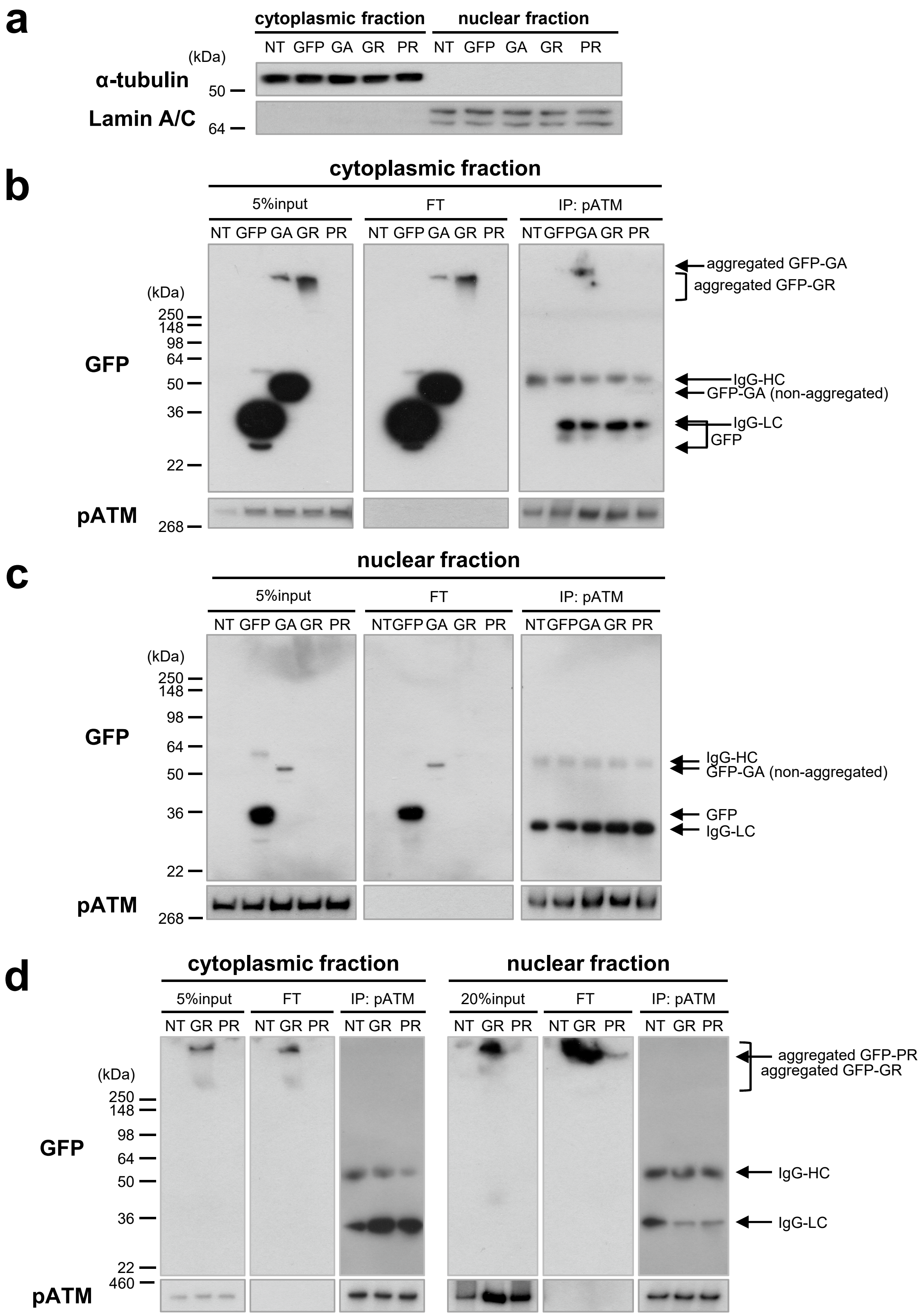
a
cytoplasmic fraction
nuclear fraction
NT GFP GA GR PR NT GFP GA GR PR
(kDa)
α-tubulin
50
Lamin A/C
64
b
cytoplasmic fraction
5%input
FT
IP: pATM
NT GFP GA GR PR
(kDa)
250
148
98
64
50
36
22
GFP
pATM
268
aggregated GFP-GA
aggregated GFP-GR
IgG-HC
GFP-GA (non-aggregated)
IgG-LC
GFP
c
nuclear fraction
5%input
FT
IP: pATM
IgG-HC
GFP-GA (non-aggregated)
GFP
IgG-LC
d
cytoplasmic fraction
nuclear fraction
20%input
NT GR PR
460
aggregated GFP-PR
aggregated GFP-GR

Suppl. Fig. 8

Supplementary Table 1: Clinical background of C9orf72 patients and control cases who provided fibroblasts

Case	Gender	Age at disease onset (years old)	Age at biopsy (years old)	Disease duration (months)	Clinical phenotype
C9-case1	female	49	51	36	ALS-FTD, bulbar
C9-case2	male	54	56	13	ALS, Spinal
C9-case3	female	66	68	26	ALS, bulbar
Ct-case1	female	n.a.	48	n.a.	n.a.
Ct-case2	male	n.a.	34	n.a.	n.a.

n.a. : not applicable

Supplementary Table 2: statistical analysis data

Figure No.	statistical method	sample		p-value
Figure 2c	2 tailed paired t test	NT-siCt		0.385
		NT-siA3		0.005
		siCt-siA3		0.048
Figure 2d	2 tailed paired t test	NT-siCt		0.283
		NT-siA3		0.012
		siCt-siA3		0.043
Figure 2g	2 tailed paired t test	siCt-siA3		0.035
Figure 2h	2 tailed paired t test	siCt-siA3		0.004
Figure 3c sense	2 tailed paired t test	A3KO S	WT NT	0.011
			WT EV	0.011
			WT S	0.018
			A3KO NT	0.011
			A3KO EV	0.011
			A3KO S +A3 rescue	0.011
Figure 3c antisense	2 tailed paired t test	A3KO AS	WT NT	0.008
			WT EV	0.008
			WT AS	0.008
			A3KO NT	0.008
			A3KO EV	0.008
			A3KO AS +A3 rescue	0.014
Figure 3d	2 tailed paired t test	sense GA: WT-A3KO		0.007
		sense GP: WT-A3KO		0.003
		sense GR WT-A3KO		0.011
		antisense GP: WT-A3KO		0.003
		antisense PA: WT-A3KO		0.049
		antisense PR WT-A3KO		0.000
Figure 4b	one-way ANOVA and Tukey's post-hoc test	WT GFP	WT GA	0.000
			WT GR	0.021
			WT PR	0.000
			A3KO GFP	0.043
			A3KO GA	0.000
			A3KO GR	0.000
			A3KO PR	0.000
		WT GA	WT GFP	0.000
			WT GR	0.895
			WT PR	1.000
			A3KO GFP	0.895
			A3KO GA	0.000
			A3KO GR	0.516
			A3KO PR	0.018
		WT GR	WT GFP	0.021
			WT GA	0.895
			WT PR	0.897
			A3KO GFP	1.000
			A3KO GA	0.000
			A3KO GR	0.037
			A3KO PR	0.000
		WT PR	WT GFP	0.000
			WT GA	1.000
			WT GR	0.897
			A3KO GFP	0.897
			A3KO GA	0.000
			A3KO GR	0.526
			A3KO PR	0.019
		A3KO GFP	WT GFP	0.043
			WT GA	0.895
			WT GR	1.000
			WT PR	0.897
			A3KO GA	0.000
			A3KO GR	0.046
			A3KO PR	0.000
		A3KO GA	WT GFP	0.000
			WT GA	0.000
			WT GR	0.000
			WT PR	0.000
			A3KO GFP	0.000

			A3KO GR	0.037
			A3KO PR	0.685
		A3KO GR	WT GFP	0.000
			WT GA	0.516
			WT GR	0.037
			WT PR	0.526
			A3KO GFP	0.046
			A3KO GA	0.037
			A3KO PR	0.864
		A3KO PR	WT GFP	0.000
			WT GA	0.018
			WT GR	0.000
			WT PR	0.019
			A3KO GFP	0.000
			A3KO GA	0.685
			A3KO GR	0.864
Figure 4d	one-way ANOVA and Tukey’s post-hoc test	WT GFP	WT GA	0.014
			WT GR	0.039
			WT PR	0.002
			A3KO GFP	0.004
			A3KO GA	1.000
			A3KO GR	0.000
			A3KO PR	0.000
		WT GA	WT GFP	0.014
			WT GR	0.000
			WT PR	0.000
			A3KO GFP	0.000
			A3KO GA	0.339
			A3KO GR	0.000
			A3KO PR	0.000
		WT GR	WT GFP	0.039
			WT GA	0.000
			WT PR	0.976
			A3KO GFP	0.967
			A3KO GA	0.157
			A3KO GR	0.000
			A3KO PR	0.000
		WT PR	WT GFP	0.002
			WT GA	0.000
			WT GR	0.976
			A3KO GFP	1.000
			A3KO GA	0.023
			A3KO GR	0.007
			A3KO PR	0.000
		A3KO GFP	WT GFP	0.004
			WT GA	0.000
			WT GR	0.967
			WT PR	1.000
			A3KO GA	0.029
			A3KO GR	0.033
			A3KO PR	0.000
		A3KO GA	WT GFP	1.000
			WT GA	0.339
			WT GR	0.157
			WT PR	0.023
			A3KO GFP	0.029
			A3KO GR	0.000
			A3KO PR	0.000
		A3KO GR	WT GFP	0.000
			WT GA	0.000
			WT GR	0.000
			WT PR	0.007
			A3KO GFP	0.033
			A3KO GA	0.000
			A3KO PR	0.707
		A3KO PR	WT GFP	0.000
			WT GA	0.000
			WT GR	0.000
			WT PR	0.000
			A3KO GFP	0.000

			A3KO GA	0.000
			A3KO GR	0.707
Figure 5 b	one-way ANOVA and Tukey's post-hoc test	Ct case, siCt	Ct case, siA3	0.975
			C9 case, siCt	0.458
			C9 case, siA3	0.001
		Ct case, siA3	Ct case, siCt	0.975
			C9 case, siCt	0.704
			C9 case, siA3	0.003
		C9 case, siCt	Ct case, siCt	0.458
			Ct case, siA3	0.704
			C9 case, siA3	0.035
		C9 case, siA3	Ct case, siCt	0.001
			Ct case, siA3	0.003
			C9 case, siCt	0.035
Figure 5 d	one-way ANOVA and Tukey's post-hoc test	Ct case, siCt	Ct case, siA3	0.999
			C9 case, siCt	0.545
			C9 case, siA3	0.000
		Ct case, siA3	Ct case, siCt	0.999
			C9 case, siCt	0.611
			C9 case, siA3	0.000
		C9 case, siCt	Ct case, siCt	0.545
			Ct case, siA3	0.611
			C9 case, siA3	0.048
		C9 case, siA3	Ct case, siCt	0.000
			Ct case, siA3	0.000
			C9 case, siCt	0.048
Figure 5 f	one-way ANOVA and Tukey's post-hoc test	Ct case, siCt	Ct case, siA3	0.904
			C9 case, siCt	0.313
			C9 case, siA3	0.000
		Ct case, siA3	Ct case, siCt	0.904
			C9 case, siCt	0.915
			C9 case, siA3	0.043
		C9 case, siCt	Ct case, siCt	0.313
			Ct case, siA3	0.915
			C9 case, siA3	0.029
		C9 case, siA3	Ct case, siCt	0.000
			Ct case, siA3	0.043
			C9 case, siCt	0.029
Figure 6b	Pearson correlation coefficient	nuclear A3signal intensity-γH2AX positivity		0.039
Figure 6d	Pearson correlation coefficient	poly-GA positivity-pATM foci positivity		0.043
Figure 7b	2 tailed paired t test	Ct-C9		0.010
Supplementary Figure 4a	2 tailed paired t test	mCh S	mCh NT	0.019
			mCh EV	0.019
			mCh-A3 NT	0.019
			mCh-A3 EV	0.019
			mCh-A3 S	0.031
	2 tailed paired t test	mCh AS	mCh NT	0.017
			mCh EV	0.017
			mCh-A3 NT	0.020
			mCh-A3 EV	0.017
			mCh-A3 AS	0.026
Supplementary Figure 4b	2 tailed paired t test	sense GA: mCh - mCh-A3		0.000
		sense GP: mCh - mCh-A3		0.001
		sense GR mCh - mCh-A3		0.007
		antisense GP: mCh - mCh-A3		0.000
		antisense PA: mCh - mCh-A3		0.000
		antisense PR mCh - mCh-A3		0.025
Supplementary Figure 5b	Pearson correlation coefficient	nuclear A3 signal intensity-nuclear FUS intensity		0.280
Supplementary Figure 5c	Pearson correlation coefficient	nuclear FUS signal intensity-γH2AX positivity		0.995
Supplementary Figure 5e	Pearson correlation coefficient	nuclear A3 signal intensity-nuclear HuR intensity		0.314
Supplementary Figure 5f	Pearson correlation coefficient	nuclear HuR signal intensity-γH2AX positivity		0.888

Supplementary Table 3: Individual immunohistochemical data of 9 controls and 16 C9orf72-cases

case	case No.	nuclear A3 intensity	γH2AX foci positivity (%)	GA positivity (%)	Nuclear pATM foci positivity (%)	Cytoplasmic pATM aggregate Positivity (%)	nuclear FUS intensity	nuclear HuR intensity
C9	1	2.0	23.9	21.9	7.6	0.3	6.3	5.1
C9	2	2.9	12.6	28.1	5.9	0.4	2.9	3.6
C9	3	3.5	21.0	16.2	37.9	2.4	5.0	3.8
C9	4	3.6	18.6	14.6	15.4	2.7		
C9	5	4.0	18.6	19.7	13.7	2.2	2.5	5.3
C9	6	4.2	17.0	31.3	14.8	1.0	4.2	3.1
C9	7	4.5	26.4	16.2	29.7	10.9	1.1	1.2
C9	8	4.6	22.9	12.7	23.3	2.5	16.6	11.0
C9	9	5.6	28.6	22.1	7.7	1.9	8.6	2.4
C9	10	6.2	20.1	11.5	10.1	1.9	8.5	6.9
C9	11	6.2	10.3	29.1	8.3	4.9	3.7	3.7
C9	12	8.2	12.0	14.0	10.9	1.8	16.1	6.0
C9	13	9.4	7.1	23.3	11.9	1.9	5.2	3.0
C9	14	9.9	15.6	9.5	21.6	1.8	6.5	7.0
C9	15	10.6	10.4	13.5	24.7	0.9	6.8	4.6
C9	16	16.9	13.0	8.3	23.4	0.6	10.0	7.6
Ct	1	2.2	13.3	0.0	10.8	1.3	2.8	2.9
Ct	2	5.6	9.5	0.0	5.7	0.0	5.0	4.8
Ct	3	8.8	7.4	0.0	4.4	0.4	1.5	4.0
Ct	4	3.8	9.1	0.0	1.1	0.0	2.9	2.0
Ct	5	5.4	5.2	0.0	11.0	0.0	4.5	3.4
Ct	6	6.9	1.3	0.0	13.3	0.4	3.1	5.9
Ct	7	6.2	12.0	0.0	9.8	0.0	7.5	3.8
Ct	8	5.1	8.6	0.0	15.3	0.7	3.2	3.5
Ct	9	18.0	3.1	0.0	4.2	1.3	6.7	4.7
